# Exploring Patient Empowerment and Health System Outcomes Associated With MyHealthNB, a Provincial Personal Health Record System: Exploratory Mixed Methods Study

**DOI:** 10.2196/87521

**Published:** 2026-04-28

**Authors:** Paula Voorheis, Stephan U Dombrowski, Frances Bruno, Carolyn Steele Gray

**Affiliations:** 1 School of Pharmacy University of Wisconsin–Madison Madison, WI United States; 2 Science of Care Institute Sinai Health System Toronto, ON Canada; 3 Institute of Health Policy, Management and Evaluation University of Toronto Toronto, ON Canada; 4 Department of Kinesiology University of New Brunswick Fredericton, NB Canada

**Keywords:** personal health records, patient portals, patient empowerment, behavior change, mixed methods

## Abstract

**Background:**

Personal health record systems (PHRs) have been introduced to support patient empowerment by giving individuals direct access to their personal health information and other key health system resources. MyHealthNB is a province-wide PHR in New Brunswick, Canada, that allows residents to view laboratory results, medication lists, immunization records, imaging reports, and a range of digital health resources. As PHRs continue to expand, it is essential to understand how PHRs like MyHealthNB impact outcomes related to patient empowerment.

**Objective:**

This study uses MyHealthNB as a case example to examine empowerment-related impacts of PHRs on citizens. Building on a conceptual framework linking patient enablement, empowerment, involvement, and engagement, the study is guided by two questions: (1) What perceived impacts of PHR use emerge across enablement, empowerment, involvement, engagement, and cost-related outcomes? (2) Which impacts of PHR use are most prevalent, how are they interrelated, and what characteristics predict variation in these impacts?

**Methods:**

An exploratory sequential mixed methods study design was used. Phase 1 involved qualitative interviews with citizens to explore perceived impacts of using MyHealthNB, which were analyzed using rapid qualitative analysis. Findings informed a Phase 2 cross-sectional survey that measured MyHealthNB users’ self-reported impacts across enablement, empowerment, involvement, engagement, and cost-related outcomes. Survey data were analyzed using descriptive statistics, *t* tests, mediation analysis, and multivariable linear regressions to examine impacts, impact pathways, and impact predictors.

**Results:**

Data from 32 interviewees and 885 survey respondents were analyzed. The qualitative analysis showed that MyHealthNB supported a progression from improved access to health information (enablement), to increased confidence (empowerment), to more active participation in health management and health care decisions (involvement and engagement). The survey analysis confirmed significant positive impacts across all 21 outcomes measured that spanned enablement, empowerment, involvement, engagement, and cost-related outcomes (*P*<.05). Mediation analyses showed that higher perceived enablement through MyHealthNB was associated with greater patient involvement and engagement, with empowerment emerging as a central linking factor. Regression models identified key predictors of MyHealthNB impacts, which included satisfaction with MyHealthNB, having a family doctor, provider support of MyHealthNB, digital literacy, and MyHealthNB use frequency.

**Conclusions:**

Exploratory, self-reported citizen data suggest that PHRs may improve outcomes related to patient empowerment, behavior change, and health system benefits. The advantages of PHR use were most prominent when individuals had access to primary care, received support from health care providers, and had confidence using digital technologies. To fully realize the promise of PHRs, implementers should invest in digital literacy support and strengthen primary care access and integration.

## Introduction

### The Rise of Personal Health Records

Over the past several decades, health care systems have increasingly embraced patient- and person-centered care, engaging citizens as active partners in their own health care [1-3]. Central to this shift is the recognition that informed, empowered patients are better equipped to manage their health and participate in their care [4-6]. Electronic personal health record systems (PHRs), also commonly referred to as patient portals, have been introduced to support this shift, providing patients with digital access to their personal health information [7-10]. PHRs generally offer individuals secure, web- or app-based access to information such as laboratory results, medication lists, imaging reports, and vaccination records, and additional features such as appointment scheduling, secure messaging, wait times dashboards, and health reminders [11,12].

### Toward a Deeper Understanding of PHR Impacts

Growing investment in PHRs has highlighted the need to understand their impact on patient-centered outcomes. A 2021 Cochrane Review examined 10 studies with 78 to 4500 participants on the effects of providing adult patients with access to electronic health records on a range of patient, patient‐provider, and health system outcomes [10]. The quality of evidence was assessed as low, and the authors suggested that future studies focus more on assessing patient empowerment and behavioral outcomes, rather than on health‐related outcomes alone [10]. This review solidified the need for more comprehensive evaluations that explore why and how PHRs influence outcomes related to patient empowerment.

### PHRs and Empowerment-Related Outcomes

**Figure 1:**
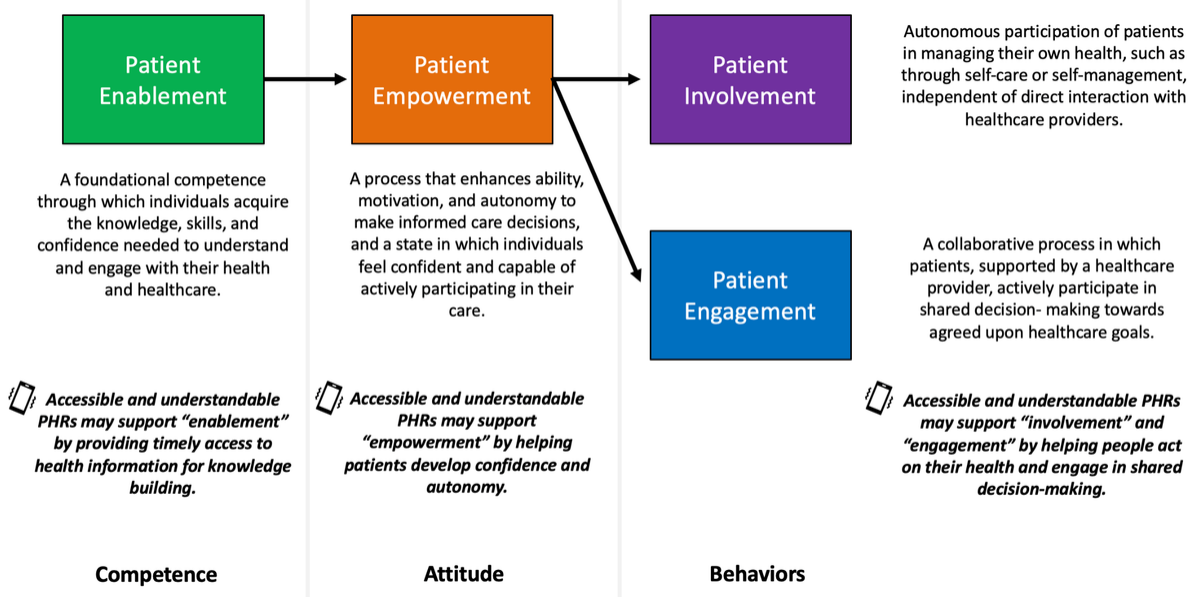
A conceptual framework of patient empowerment and related concepts (adapted from Hickmann et al, 2022 [16]). PHR: personal health record.

To understand how PHRs affect empowerment-related outcomes, it is essential to first define patient empowerment and distinguish it from related concepts such as patient enablement, engagement, and involvement [13-16]. A 2022 systematic review proposed a continuum that distinguishes and connects these interrelated constructs [16]. In this continuum, “patient enablement” refers to the foundational competence through which individuals acquire the knowledge, skills, and confidence needed to understand and engage with their health and health care. Enablement fosters “patient empowerment,” which is both a process that enhances a person’s ability, motivation, and autonomy to make informed health decisions and a state in which individuals feel confident and capable of actively participating in their care. Empowered patients may then demonstrate 2 types of behavioral change: “patient involvement,” where individuals autonomously manage their own health (eg, through self-care or self-management), independent of direct interaction with health care providers; and “patient engagement,” where patients actively participate in health care behaviors in collaboration with a health care provider using shared decision-making. Figure 1 illustrates these conceptual distinctions.

PHRs may support each stage of this progression by providing timely and accessible health information that supports individuals in building foundational knowledge (patient enablement), developing confidence and autonomy (patient empowerment), and engaging in independent health actions and collaborative decision-making (patient involvement and engagement) [7,17]. The conceptual distinctions in Figure 1 provide the foundation for future research to identify relevant measures of each construct and to better understand how PHR use may influence different aspects of these concepts.

### Personal and Health System Cost Outcomes

In addition to empowerment-related outcomes, the impact of PHRs on personal and health system usage is a key area of interest in both the literature and public discourse [18-20]. From the citizen perspective, PHRs may help decrease time spent finding and organizing health information, the number of calls or visits to a health care provider to access information, and even emergency room visits [18,19]. Understanding citizens’ perceptions of personal and system-related time and usage savings is important, as these experiences may also have broader implications for system costs and efficiency.

### PHRs in Canada

Canada has made substantial progress in implementing PHRs, with many provinces and territories developing systems to provide citizens with timely, interoperable access to their health information. Examples of province-wide PHRs include MyHealthNB in New Brunswick, YourHealthNS in Nova Scotia, Health Booklet in Quebec, MySaskHealthRecord in Saskatchewan, MyHealth in Alberta, and Health Gateway in British Columbia. A 2024 report by Canada Health Infoway found that 89% of Canadians want access to their health records via web [21]. Public demand for PHR access creates an opportunity to explore how these systems are perceived and experienced by users, with a focus on patient-centered outcomes. In particular, understanding whether PHRs help citizens become more enabled, empowered, and active in their health and health care can support better design and implementation.

### New Brunswick’s PHR System: MyHealthNB

**Figure 2:**
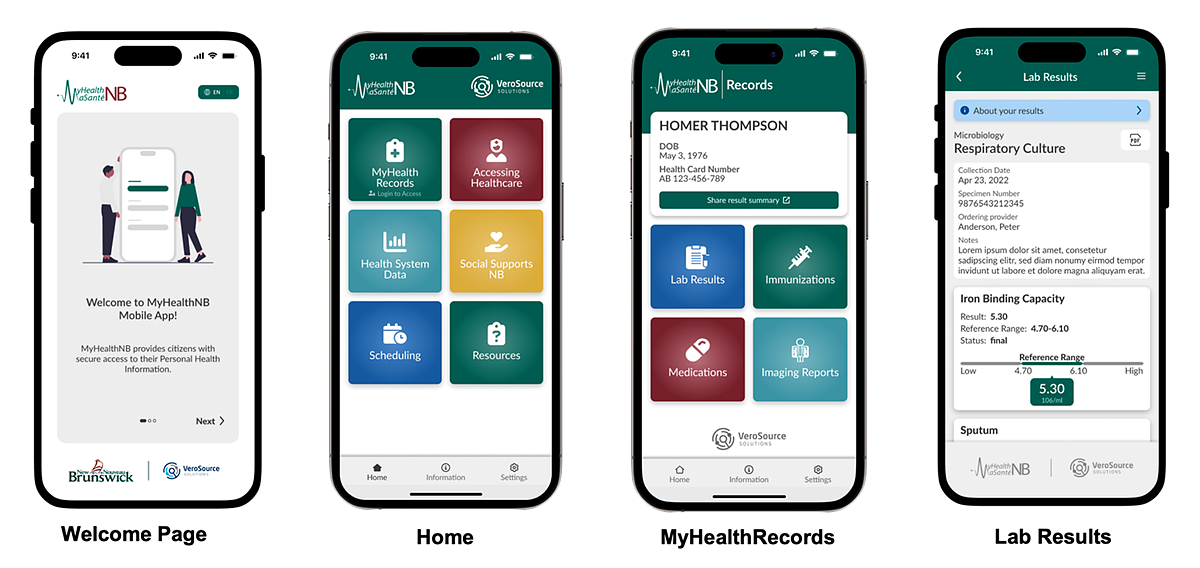
Images from MyHealthNB at the time of the study.

MyHealthNB is New Brunswick’s province-wide PHR system, available through a mobile app or online web portal at the time of this study [22]. MyHealthNB was first made available to the public in early 2020 in response to the COVID-19 pandemic, providing citizens with access to their COVID-19 test results. Subsequent changes to the information available through MyHealthNB between its launch and the start of this study are outlined in Multimedia Appendix 1. During this study, MyHealthNB’s home page connected residents to a range of health-related resources, including access to their personal health information through “MyHealthRecords.” Additional resources included health system data (eg, wait times information), guidance on how to access care (eg, how-to links for finding services), information on social supports (eg, programs and services), self-scheduling tools (eg, x-ray booking and eVisitNB), and general health system resources (eg, links to regional health authority websites and mental health supports). Within “MyHealthRecords,” users could view their personal laboratory results, medications, immunizations, and imaging reports. At the time of this study, MyHealthNB did not include patient–provider messaging or links to educational information related to test results, tests, or medications. Screenshots of MyHealthNB and its key features are shown in Figure 2. More detailed information about the features and content included in MyHealthNB since its initial launch until the study period can be found in Multimedia Appendix 1.

### Research Questions

This study uses MyHealthNB as a case example to examine empowerment-related and behavior change impacts (eg, involvement and engagement behaviors) of PHR systems on citizens. The study is guided by the following questions:

1. What are the perceived impacts of MyHealthNB across the domains of enablement, empowerment, involvement, engagement, and cost-related outcomes (eg, personal and health system costs) according to New Brunswick citizens?
2. Which perceived impacts of MyHealthNB are most prevalent among MyHealthNB users? How are these impacts interrelated, and what characteristics (ie, demographic factors, digital literacy, health conditions, health care attachment, and patterns of MyHealthNB use) are associated with variation in these impacts?

By answering these questions, the study aims to generate actionable insights into how PHRs support patient-centered outcomes related to patient-empowerment, and to inform the future design and evaluation of PHRs.

## Methods

### Study Design and Approach

An exploratory sequential mixed methods design was used, beginning with qualitative data collection to identify perceived impacts, followed by a quantitative phase to broadly measure the identified impacts and their predictors [23].

This study was part of a broader developmental evaluation aimed at assessing and improving the design, implementation, and impact of MyHealthNB in New Brunswick. Developmental evaluation is an approach used during the early stages of an innovation to understand what is effective and what requires adjustment, particularly for complex and evolving programs like MyHealthNB [24,25]. The purpose of developmental evaluation is to learn and adapt the intervention as it is being delivered, ensuring evaluation methods are purposefully selected to allow for rapid transformation in innovation development [24,25]. Unlike traditional evaluation models, a developmental evaluator serves as a collaborative partner rather than an external assessor, embedding evaluation within the innovation process. In this study, the lead evaluator (PV), in support of the academic team (SUD and CSG), worked closely with VeroSource Solutions Inc. (the developer), the New Brunswick Department of Health (the implementer), and Canada Health Infoway (a national supporter of implementation) to build the evaluation strategies. One of the central objectives of the overall developmental evaluation was to understand and measure the impacts MyHealthNB was having on citizens.

### Reflexivity

This study was informed by a relativist ontological stance, acknowledging that reality is subjective and shaped by individuals’ lived experiences [26]. Epistemologically, the research was guided by a blend of constructivism and pragmatism [27]. Constructivism supported the co-construction of meaning between researchers and participants during the qualitative phase, while pragmatism underpinned the study’s emphasis on producing actionable knowledge to inform ongoing PHR implementation. Consistent with this orientation, the study used a naturalistic and practice-oriented approach to inquiry, using descriptive and rapid analyses to generate context-sensitive insights that could support real-time learning and improvement for MyHealthNB [28].

As consistent with established developmental evaluation methods, researchers worked closely with those involved in implementing MyHealthNB. While this proximity supported a strong understanding of context while providing opportunities to provide evaluation feedback to implementers (a core aim of developmental evaluation [24,25]), steps were taken to reduce the risk of bias throughout data collection, analysis, and interpretation. All interviews were conducted by members of the research team only, without the presence of the health system or implementation partners. Qualitative coding and quantitative analyses were also completed solely by the research team, and partners were not involved in analytic decisions or in determining which findings were reported. Throughout the study, the research team engaged in ongoing reflection to discuss partnership dynamics and reflect on the positive and negative findings.

### Phase 1: Qualitative Interviews

#### Participants and Recruitment

New Brunswick citizens were recruited in both English and French to participate in semistructured interviews exploring their experiences and perceptions of MyHealthNB and online access to health information. Recruitment was conducted through established patient and caregiver groups affiliated with the province’s regional health authorities, which distributed a recruitment email to their members. The email described MyHealthNB, the study purpose, and what participation would involve. This approach was supplemented by snowball sampling to support demographic and experiential diversity in the sample, including variation in age, race, gender, geographic location, and MyHealthNB usage frequency. Participants were eligible if they were 19 years of age or older and able to complete the interview in English or French. Prior experience with MyHealthNB was not required. Participants without prior experience were instructed that they would be guided through MyHealthNB by the interviewer and would be asked to reflect on their initial perceptions of MyHealthNB and its potential role in their health care journey. Prior use was not required because the broader developmental evaluation aimed to gather citizen feedback on the design, implementation, and impact of MyHealthNB. Including both users and nonusers (eg, those who had attempted but were unable to access the platform, those who had chosen not to use it, or those who were unaware of it) enabled the study to capture both realized and potential impacts of MyHealthNB on citizens’ health care experiences. The final sample size was guided by the principle of conceptual depth, with data collection concluding once sufficient richness and relevance to the research objectives had been achieved [29]. Conceptual depth was assessed through iterative review of interview data, focusing on the recurrence of key concepts across participants and the ability of the data to explain how and why MyHealthNB influenced citizens’ experiences. Ongoing analytic discussions within the research team (PV and CSG) were used to assess whether additional interviews were yielding new insights and when data collection should stop.

#### Data Collection

Data were collected using semistructured interview guides and a preinterview survey in either French or English, depending on participant preference [30]. Interviews were conducted via Zoom or phone and lasted approximately 45 to 60 minutes. The interview guides were piloted and refined based on input from coauthors and partners from VeroSource Solutions Inc. and the New Brunswick Department of Health. Interview guides differed based on prior use of MyHealthNB. For participants with prior MyHealthNB experience, interviews explored past experiences navigating health information within the New Brunswick health care system, how participants became aware of and adopted MyHealthNB, their current engagement with the platform, perceived impacts on their care, likes and dislikes, and recommendations for future development. During these interviews, the interviewer could share their screen to display a mock version of MyHealthNB to facilitate discussion of specific features. For participants without prior MyHealthNB experience, interviews focused on past experiences navigating health information within the New Brunswick health care system, awareness of MyHealthNB, and initial interests or concerns about the platform. The interviewer then shared their screen to present a mock version of MyHealthNB, and participants were asked to discuss their perceptions of the platform and the potential impacts on their care if they were to adopt different features. Participants were subsequently asked about likes, dislikes, and recommendations for the future of MyHealthNB. All interviews were audio-recorded and transcribed with an accuracy check using Zoom’s automated recording and transcription feature. Translation was performed with the support of a fluent bilingual French–English speaking team member (FB), and using Microsoft Office Translator in Microsoft Word. The preinterview survey gathered information on participants’ demographics and MyHealthNB usage, the results of which are presented later.

#### Data Analysis

Descriptive statistics were generated to characterize the interview sample. The interview data were analyzed using rapid qualitative analysis in Microsoft Word [31-33]. Rapid qualitative analysis is an action-oriented approach designed to efficiently generate insights that inform decision-making in dynamic or time-sensitive contexts [31-33]. Following established steps for rapid qualitative analysis, the lead researcher (PV) reviewed the interview transcripts and audio recordings to create summary documents that captured key points from each interview. These summaries were structured according to predefined domains aligned with the objectives of the broader developmental evaluation, which focused on identifying design-related facilitators and barriers, implementation-related facilitators and barriers, and the perceived impacts of MyHealthNB. This study focused on perceived impacts. When relevant, illustrative quotes were included alongside summary points. Summary data from each interview were compiled into a larger matrix to allow for cross-interviewee comparison of perceived impacts. Within the matrix, points related to enablement, empowerment, involvement, and engagement were labeled according to previously outlined definitions [16]. The matrix was used to organize the data into a concise, accessible format that could support descriptive interpretation and application. The matrix template was reviewed by CSG.

### Phase 2: Integration of Qualitative and Quantitative Steps

The preliminary findings from the qualitative interviews in Phase 1 were used to inform both the sampling strategy and instrument design for the quantitative survey.

Regarding the sampling strategy, interviewees highlighted several population groups that may experience unique impacts from MyHealthNB, including individuals enrolled in school, older adults with caregivers, newcomers to New Brunswick, those living with chronic conditions (eg, diabetes and cancer), individuals involved in community or recreational groups, persons with disabilities, and members of racial minorities or Indigenous communities. In response, PV conducted targeted outreach to publicly available contacts associated with organizations representing these groups to enhance representation in the quantitative sample.

Regarding the instrument design, qualitative insights were used to ensure that survey questions reflected the diverse ways in which MyHealthNB may have impacted participants and identified contextual factors that shaped those experiences. Survey items were developed to capture self-perceived impacts across patient enablement, empowerment, involvement, engagement, and cost-related outcomes. Interview findings also informed the inclusion of additional predictors such as whether respondents were new to New Brunswick, their education level, access to a family doctor, presence of a chronic condition, extent of MyHealthNB use (eg, use of homepage), and whether their providers encouraged use of MyHealthNB. A full description of survey measures is included below and in Multimedia Appendix 1. The alignment between qualitative insights and survey design ensured that the quantitative phase was grounded in the realities of patient experience.

Although validated instruments such as the Patient Activation Measure, Health Empowerment Scale [34] were considered during the design of the quantitative phase, these tools were ultimately not adopted because they were fully responsive to the specific impacts MyHealthNB was intended to have. Given the developmental evaluation focus, survey items were designed to capture PHR-specific perceived impacts across domains of enablement, empowerment, involvement, engagement, and system costs. The final survey was developed iteratively based on qualitative findings, established conceptual distinctions in the academic literature [10,16,35], and measures used in similar evaluations of PHRs in other Canadian jurisdictions [19,20].

### Phase 3: Quantitative Cross-Sectional Survey

#### Participants and Recruitment

Individuals were eligible if they resided in New Brunswick and were 19 years of age or older. The recruitment strategy aimed to obtain a broadly representative sample of the provincial population. Outreach efforts were guided by patient and caregiver advisory groups and informed by insights from the qualitative interviews. Targeted email invitations were sent to organizations representing priority groups, including students, older adults and their caregivers, newcomers to New Brunswick, individuals with chronic health conditions, community and recreational group participants, persons with disabilities, and members of racialized or Indigenous communities. These organizations distributed the survey invitation through their existing mailing lists and communication channels. Additionally, a survey invitation was posted on the main landing page of the MyHealthNB website. Participation was voluntary and self-selected, which may have resulted in greater participation from individuals who were more digitally engaged or had more notable experiences with MyHealthNB. All recruitment materials and the survey were available in English and French.

#### Data Collection

An online survey administered through Qualtrics was used to collect data. As previously outlined, survey measures were developed specifically for this developmental evaluation and, therefore, were not subjected to formal psychometric testing. Construct validity was not assessed using methods such as factor analysis or hypothesis testing. Instead, measures were designed to maximize content relevance and interpretability through qualitative grounding, theoretical alignment, and codevelopment with system partners. Many constructs were assessed using single-item measures, which limited the ability to assess internal consistency and may increase susceptibility to measurement error or response bias. Quantitative findings should be interpreted as exploratory indicators of perceived impact rather than precise estimates of underlying constructs. The implications of these measurement limitations for interpretation are discussed further in the Limitations section.

To provide feedback on the impact of MyHealthNB, respondents were required to self-identify as users of MyHealthNB. MyHealthNB users were defined as those who reported using the MyHealthNB to access their personal health information, to help someone else access their personal health information (eg, laboratory results, immunizations, medication lists, and imaging reports), or to access general information about the health system (eg, wait time dashboards) or other health system links (eg, x-ray scheduling or eVisitNB). Respondents who indicated they had not used MyHealthNB were classified as nonusers and were directed to a different survey module that did not include questions about the impacts of MyHealthNB. The measures used to assess self-reported impacts of MyHealthNB and key descriptive variables are detailed below.

#### Dependent Variables: Impact Measures

Self-reported impact measures captured in the survey aligned with the domains of patient enablement, patient empowerment, patient involvement, patient engagement, and personal and health system costs. These measures were informed by conceptual distinctions and findings provided in the academic literature [10,16,35], surveys conducted in other Canadian jurisdictions [19,20], and descriptions and examples provided by interviewees from the qualitative phase. All measures were codeveloped, piloted, and finalized with partners at the New Brunswick Department of Health and VeroSource Solutions, ensuring both conceptual alignment and practical relevance to PHR implementation. Table 1 outlines the measures and scales used.

**Table 1:** Survey measures for self-reported impacts from MyHealthNB.

Domain	Question prompt and scale	Measures
Patient enablement	Prompt of “How has having access to your personal health records through MyHealthNB impacted the following?” with a 5-Point Likert Scale including, “Significantly Improved (5)”, “Somewhat Improved (4)”, “No Change (3)”, “Somewhat Worsened (2)”, and “Significantly Worsened (1)”	Ease in accessing your health information (eg, ability to find a specific health record) Awareness of your health status (eg, awareness of your results, vaccination status, or medication history) Understanding of your health (eg, understanding why you are experiencing a certain issue or why you need a specific treatment) Control over your health information (eg, the ability to identify errors in your record or share your health information with whom you want to)
Patient empowerment	Prompt of “Rate your level of agreement with the following statement” with a 5-Point Likert Scale including, “Strongly agree (5)”, “Somewhat agree (4)”, “Neither agree nor disagree (3)”, “Somewhat disagree (2)”, and “Strongly disagree (1)”	“MyHealthNB has empowered me to take a more active role in my health and health care by giving me greater access, control, and understanding of my health information.” “MyHealthNB has raised concerns, frustration, and stress for me because of the ongoing challenges I face in accessing, managing, and understanding my health information.”
Patient involvement	Prompt of “How has having access to your personal health records through MyHealthNB impacted the following?” with a 5-Point Likert Scale including, “Significantly Improved (5)”, “Somewhat Improved (4)”, “No Change (3)”, “Somewhat Worsened (2)”, and “Significantly Worsened (1)”	Engagement in your health care (eg, doing preventative screenings, scheduling follow up appointments, refilling prescriptions, managing an ongoing issue) Engagement in your health (eg, making healthier decisions, setting goals related to your health)
Patient engagement	Prompt of “How has having access to your personal health records through MyHealthNB impacted the following?” with a 5-Point Likert Scale including, “Significantly Improved (5)”, “Somewhat Improved (4)”, “No Change (3)”, “Somewhat Worsened (2)”, and “Significantly Worsened (1)”	Preparedness for visits with a health care provider (eg, having a plan for a concern, result, or treatment you want to discuss) Communication with a health care provider (eg, being able to discuss a concern, result, or treatment with a provider) Ability to be a more active participant in health care decision-making (eg, collaborating with a provider to choose the best treatment or next step) Preparedness for a health emergency or unexpected health event (eg, ability to quickly access what you need to share during an emergency) Early detection and action (eg, more timely seeking and addressing an abnormality) Ability to avoid taking a test or treatment you don't need or want (eg, avoiding a duplicate test or treatment) Efficiency of your health care appointments (eg, having more time to talk about what matters to you or getting treatment faster because you know your medical history) Coordination of your health care (eg, ensuring all of your health care providers are on the same page) Overall quality of your health care appointments (eg, satisfaction with your overall experience with visiting a provider)
Personal and health system cost	Prompt of “How has having access to your personal health records through MyHealthNB impacted the following?” with a 5-Point Likert Scale including, “Significantly Decreased (5)”, “Somewhat Decreased (4)”, “No Change (3)”, “Somewhat Increased (2)”, and “Significantly Increased (1)”	Time spent finding and organizing your health information (eg, time spent retrieving your health results from different providers or clinics) Number of calls you have to make to a health care provider to get health information (eg, information about test results, vaccine records, medication information, and treatment history) Number of times you travel to a health care provider to get health information (eg, travelling to receive a result) Number of times you visit the emergency room

#### Independent Variables: Other Measures

Other self-reported measures used in the study assessed respondent demographics (ie, age, race, gender, location in New Brunswick, residence length in New Brunswick, language, and education level), digital abilities (ie, digital literacy and digital health navigation ease), health status (ie, chronic condition status), health care attachment (ie, family doctor attachment status), and MyHealthNB usage (ie, MyHealthNB access format, satisfaction, usage frequency, home page usage, and provider support satisfaction). These are fully defined and outlined in Multimedia Appendix 1.

### Data Analysis

Data were analyzed using RStudio (RStudio, Inc) and Microsoft Excel. Survey responses were analyzed without weighting, as the primary objective was to examine perceived impacts among users rather than to generate population-representative estimates. Implications of self-selection and underrepresentation of certain groups are addressed in the Limitations section. Analysis proceeded in 4 sequential phases. In the first phase, descriptive statistics were generated to characterize the survey sample. Survey sample demographics were compared with provincial benchmarks from the 2021 Canadian Census and 2024 New Brunswick Health Council to assess representativeness. In the second phase, descriptive and inferential analyses were conducted to assess perceived impacts of MyHealthNB across 21 self-reported outcomes related to enablement, empowerment, involvement, engagement, and costs. For each outcome, means, SDs, 95% CIs, and one-sample *t* tests against the neutral midpoint of 3 were calculated. Likert-scale distributions were also summarized using frequencies and proportions across all 5 response options to contextualize patterns of perceived improvement, neutrality, or decline. In the third phase, regression-based mediation analyses were conducted to explore whether associations between patient enablement and downstream outcomes were consistent with hypothesized pathways involving empowerment and stress. Composite scores were calculated for enablement, involvement, engagement, and cost-related outcomes by averaging theoretically aligned survey items, and internal consistency was assessed. Empowerment and stress were measured using single-item measures. For each proposed mediator (empowerment and stress), regression models examined associations among enablement, the mediator, and each outcome (engagement, involvement, and cost-related outcomes). Outcomes were first regressed on enablement alone and then on both enablement and the proposed mediator to assess whether the mediator was associated with the outcome and whether both variables remained significant in combined models. Given the cross-sectional design, mediation analyses were intended to explore theory-consistent patterns of association rather than to test formal causal mediation or estimate indirect effects. Patterns in which enablement predicted both the mediator and the outcome, and both remained significant in combined models, were interpreted as consistent with partial mediation. In the fourth phase, multivariable linear regression analyses were conducted to identify predictors of variation in the 21 different impact scores. Independent variables included the 16 other measures outlined in Multimedia Appendix 1. Each impact dependent variable was modeled separately, and standardized beta coefficients, *P* values, and adjusted *R*^2^ values were calculated. Multicollinearity was assessed by calculating variance inflation factors (VIFs) for the regression model examining MyHealthNB’s impact on ease of accessing health information, which included the full set of predictors used across analyses. Each predictor was regressed on all remaining predictors, and VIFs were calculated using VIF = 1/(1−*R*^2^). The regression analyses were not stratified by MyHealthNB use type (eg, using MyHealthNB to access one’s own personal health information, using MyHealthNB to support others in accessing their health information, or using MyHealthNB to seek general health system information) because these use types reflected overlapping, task-based behaviors rather than stable user groups. Many respondents reported multiple, dynamic forms of use. Additionally, although the conceptual framework suggests directional relationships among enablement, empowerment, involvement, and engagement, the cross-sectional design does not support assumptions of temporal ordering or causal inference. We therefore did not conduct mediation analyses and instead focused on describing associations between predictors and perceived impacts. Full descriptive and inferential statistical outputs for all phases of the analysis are provided in Multimedia Appendix 1.

### Ethical Considerations

The study was reviewed and approved by the Research Ethics Boards at the University of New Brunswick, Sinai Health System, and University of Toronto (research ethics board 2023-169). Interview participants provided written consent using email or verbal consent at the start of the Zoom interview. Interview participants were remunerated with a gift card worth US $30 and agreed that at no point in any publications or oral proceedings would they be identified. Survey participants provided consent online using Qualtrics. At the end of the survey, participants were asked if they wanted to enter their name into a raffle to receive 1 of 10 gift cards. If they said “yes,” they were taken to another page where they were asked to enter their name and email address. Names and email addresses were in no way linked to their previous survey responses.

## Results

### Participants

In the qualitative phase, a total of 32 participants were interviewed between April and August 2024 to discuss the perceived impacts of MyHealthNB. Among interviewees, 27 out of 32 (84%) reported using MyHealthNB to access their own personal health information, 17 out of 32 (53%) reported using MyHealthNB to help someone else access health information, and 3 out of 32 (9%) reported no prior use of MyHealthNB. In the quantitative phase, 1160 New Brunswick residents completed the survey between November 27, 2024, and January 11, 2025. Of the 969 respondents who answered the question about prior use, 885 (91%) reported having used MyHealthNB and are included in the analyses below. Survey respondents could indicate multiple reasons for use: 771 out of 885 (87%) reported using MyHealthNB to access their own personal health information, 274 out of 885 (31%) reported using MyHealthNB to help someone else access health information, and 233 out of 885 (26%) reported using MyHealthNB to find general health system information (eg, wait times dashboards) or access related services (eg, x-ray scheduling or eVisitNB). The number of respondents included in each impact analysis is reported alongside the corresponding statistical outputs in Multimedia Appendix 1.

Table 2 presents the participant characteristics for both the interview and survey samples, alongside comparable population data from New Brunswick. The interview sample included a diverse mix of ages, races, genders, geographic locations, and languages. Males were underrepresented in the interview sample, though several female participants spoke on behalf of their male partners. The interview sample showed a similar distribution to the survey sample. The survey sample was broadly representative of the New Brunswick population in terms of age, race, language, location, family doctor attachment status, and chronic health conditions, but underrepresented males and individuals with lower education levels.

**Table 2:** Interview and survey participant characteristics.

Characteristics				Interview sample (n=32), n (%)		Survey sample (n=885), n (%)		Population data, %
**Demographics**								
	**Age (years)**							
		19-34	9 (28)		197 (22)		21	
		35-44	4 (13)		158 (18)		15	
		45-54	5 (16)		156 (18)		16	
		55-64	4 (13)		161 (18)		20	
		65-74	9 (28)		158 (18)		17	
		75-84	3 (9)		45 (5)		8	
		85+	0 (0)		2 (<1)		3	
		No response	0 (0)		8 (1)		NR^a^	
	**Race**							
		Racial majority (White)	28 (88)		770 (87)		94	
		Racial minority	2 (6)		89 (10)		6	
		Prefer not to say	1 (3)		14 (2)		NR	
		No response	1 (3)		12 (1)		NR	
	**Gender**							
		Male	8 (25)		217 (25)		49	
		Female	23 (72)		651 (74)		51	
		Trans or nonbinary	0 (0)		3 (<1)		NR	
		Prefer not to say	1 (3)		2 (<1)		NR	
		No response or other	0 (0)		12 (1)		NR	
	**Location**							
		Major health zone (zone 1-3)	29 (91)		690 (77)		76	
		Rural health zone (zone 4-7)	3 (9)		137 (15)		24	
		No response or other	0 (0)		58 (7)		NR	
	**Language**							
		English only	20 (63)		595 (67)		58	
		French and English	12 (38)		238 (27)		34	
		French only	0 (0)		36 (4)		8	
		No response or other	0 (0)		16 (2)		NR	
	**Residence length**							
		0-3 years	NR		37 (4)		NR	
		3-10 years	NR		115 (13)		NR	
		10-20 years	NR		98 (11)		NR	
		20+ years	NR		619 (70)		NR	
		No response or other	NR		16 (2)		NR	
	**Education level**							
		No advanced degree/certificate	NR		198 (22)		49	
		Advanced degree/certificate	NR		669 (76)		51	
		No response or other	NR		18 (2)		NR	
**Digital literacy**								
	**Web confidence**							
		Very confident	27 (84)		587 (66)		NR	
		Somewhat confident	4 (13)		178 (20)		NR	
		Not very confident	1 (3)		15 (2)		NR	
		Not confident at all	0 (0)		5 (1)		NR	
		No response or other	0 (0)		6 (1)		NR	
	**Digital health navigation**							
		Very easy	7 (22)		200 (23)		NR	
		Somewhat easy	17 (53)		226 (22)		NR	
		Somewhat challenging	7 (22)		169 (19)		NR	
		Very challenging	1 (3)		47 (5)		NR	
		Have not attempted	0 (0)		115 (13)		NR	
		No response or other	0 (0)		138 (16)		NR	
**Health condition**								
	**Ongoing health condition**							
		Yes	NR		663 (75)		68	
		No	NR		197 (22)		32	
		No response or other	NR		15 (2)		NR	
**Health care attachment**								
	**Family doctor attachment status**							
		Yes	NR		642 (73)		76	
		No	NR		202 (23)		24	
		No response or other	NR		41 (5)		—^b^	
**MyHealthNB usage**								
	**Access format**							
		Web only	12 (38)		458 (52)		NR	
		App only	6 (19)		120 (14)		NR	
		Web and App	11 (34)		283 (32)		NR	
		No response	3 (9)		24 (3)		NR	
	Satisfaction score (Max=4), mean score (n)		3.41 (29)		3.42 (831)		NR	
	**Usage frequency**							
		Low	17 (53)		487 (55)		NR	
		Medium	12 (38)		266 (30)		NR	
		High	0 (0)		80 (9)		NR	
		No response	3 (9)		52 (6)		NR	
	**Home page use**							
		Yes	NR		399 (45)		NR	
		No	NR		413 (47)		NR	
		No response	NR		73 (8)		NR	
	**Provider support**							
		Very satisfied	NR		182 (21)		NR	
		Somewhat satisfied	NR		148 (17)		NR	
		Neutral	NR		363 (41)		NR	
		Somewhat dissatisfied	NR		68 (8)		NR	
		Very dissatisfied	NR		63 (7)		NR	
		No response	NR		61 (7)		NR	

^a^NR: not recorded.

^b^Not available.

### Qualitative Results

#### Overview

Interviews with New Brunswick citizens revealed that MyHealthNB was seen as more than a tool for viewing results and personal health history; it served as a catalyst for behavior change and relational improvements in health care. Interview participants traced a clear progression from gaining access to health information (ie, enablement), to feeling more confident and in control (ie, empowerment), to actively participating in care (ie, engagement and involvement), and noted the emotional and cognitive complexity that accompanied this shift. The sections below outline the foundational ways MyHealthNB impacted participants.

#### From Enablement to Action: How Patients Used MyHealthNB to Manage Their Health

Interview participants described enablement as MyHealthNB’s foundational value, providing timely, understandable access to personal health information:

I had all the information in the palm of my hand, whereas before, I was relying on my doctor and hoping that you would talk to a doctor because doctors as so busy. It’s so nice to be able to get the information a lot quicker…

Interviewee #9

I used to get pretty archaic looking lab print outs emailed to me. I had to work hard to figure it out and understand it. So, the difference now is that it’s much more intuitive to understand what you’re looking at and make sense of it.

Interviewee #11

This enablement often initiated a chain reaction toward empowerment and behavior change, particularly in how patients prepared for appointments:

For me [MyHealthNB] is awesome, because when I go to the hospital [for cancer treatment] I am able to check my blood results and compare my results from last time. If my blood results are fine and they say to go ahead, I can compare and say I also should be good to go ahead this time. I like to understand the lab results… so it is kind of educational as well at the same time… because you can go further and do some research and talk to your doctor…

Interviewee #30

#### Empowerment Through MyHealthNB: Balancing Feelings of Confidence and Concern

Interview participants consistently described MyHealthNB as a powerful facilitator of patient empowerment, particularly through its ability to enhance feelings of control and peace of mind. For many, access to timely health information allowed them to approach medical appointments with greater confidence and clarity, reducing feelings of helplessness:

One big thing is to have that information sooner, which gives you peace of mind. Sometimes when being tested for something your mind starts thinking crazy stuff. So peace of mind is one big thing.

Interviewee #9

[With MyHeathNB] I could be emotionally and informationally prepared for the appointments to ask specific questions. For people with cancer the wait for results is crucial. Some people can go through depression. For me, my morale is very solid, but I can understand how waiting is hell.

Interviewee #30

I feel like I am in control, which I should be. [MyHealthNB] gives me peace of mind. I am not worried about my health as much and, as best as I can, I am in control… I feel like I can understand everything, and I know if it’s not my doctor who can deal with it, my dietician can deal with it. It gives me the information I need.

Interviewee #1

At the same time, many participants emphasized that empowerment through information from MyHealthNB was not without emotional and cognitive complexity. While MyHealthNB provided reassurance and autonomy, it also introduced new challenges that could generate confusion, worry, or stress:

Yeah, for me, it's like, if it's not in the green, then that worries me. I'm like, ‘Oh, am I deficient in XYZ?’ I just don't want to look at [MyHealthNB] anymore. I started to go into that kind of spiral of looking it up online and trying to figure out what everything means.

Interviewee #29

My poor mom, the condition we have got affects every body system, so she’s got so many things going on. She doesn’t know which doctor she’s supposed to ask which question to, and I don’t want to suggest it to her these days because she very anxious normally. So to see all these results when things get out of whack makes her very anxious.

Interviewee #15

Although viewing results in MyHealthNB sometimes led to worry and uncertainty, many participants did not see these feelings as contradictory to empowerment. Rather, they viewed them as an inherent part of the empowerment process, recognizing that emotional engagement, both positive and negative, is often intertwined with taking a more active role in one’s health:

I recently saw something that was usually in the normal range was out of range, and I googled it to find out it had to do with my liver. This was something new. My doctor has a nurse who speaks to you on the phone first, and I told her about this. Immediately the doctor came on and talked to me about it. I don’t know if they ever would have looked at it if I had not seen it. Being able to go on MyHealthNB meant that I could advocate for myself.

Interviewee #12

It may create some anxiety in people if they're not used to [seeing their records], and if it's a new problem. But people with chronic problems, when they see their doc, and they've been getting tests for years, they know what's good, they know what's bad.

Interviewee #13

#### Patient Involvement: MyHealthNB Allows Patients to Take Control of Their Own Health

Participants described how MyHealthNB supported involvement in their own health in a context where primary care follow-up and communication of test results are often limited. By providing direct access to laboratory results, MyHealthNB allowed patients to review their information, track changes over time, and take action between appointments:

I went in [MyHealthNB] and checked my blood test and I saw my blood sugar was a little bit high… So, I said okay, give yourself 6 months and get the sugar out of your diet and try to make a few lifestyle changes to see if you can bring it around…You don’t want it to get too far out of line and I certainly don’t want to have to take any more medication.

Interviewee #31

I am a Type 1 Diabetic, and I am very involved in my own care. I see [MyHealthNB] being a ‘game changer’. And I hate that terminology, but this is really a game changer for patients to take control of their own health and to learn about their own bodies and what they need to do to improve their results.

Interviewee #1

#### Patient Engagement: MyHealthNB Can Drive Impact Through Enhancing Relational Care

Interview participants emphasized that MyHealthNB helped them engage more actively and collaboratively with their health care providers. Access to health information allowed patients to prepare questions, guide discussions, and engage more collaboratively during clinical encounters when providers were receptive to patient involvement:

What I tend to do is highlight [my medical reports] and comment on the things I want answers to. Then I talk to my clinical nurse and ask, “okay, this is that?”. I feel very fortunate. If I didn’t have the results to download [through MyHealthNB], I wouldn’t be able to ask those questions.

Interviewee #14

I hope physicians understand that [MyHealthNB] will make it easier on them because now we are partners. I’ve got the information, they’ve got the information... If my blood tests are bad because of my diet in the last year… do I know enough how to fix my own diet? If not, can they send me to a dietician?

Interviewee #1

While MyHealthNB enabled many participants to take greater initiative in clinical conversations, their ability to fully engage often depended on how providers responded. Supportive providers reinforced the shift toward partnership and shared decision-making, whereas dismissive or resistant attitudes risked undermining it:

I met [my doctor] in the hospital when I was visiting a friend… I told him about MyHealthNB and its great people can advocate for themselves and he said, ‘It’s not really good... people going to Dr. Google… you know you’d misinterpret your results”. He wasn’t very impressed by us having our records in MyHealthNB.

Interviewee #16

I thought that I should talk to my doctor about [MyHealthNB] and she was not very receptive of it and said ‘oh you don’t need that’. She didn’t tell me about it and it was not something of interest to her… We don’t have that close of a relationship and I know they are very taxed and their time is precious.

Interviewee #28

#### System Usage Impacts: Patients Perceive Reduced Burden, Not Increased Demand

While some interviewees acknowledged that they had heard of providers voicing concerns that MyHealthNB could increase the health care system burden through more calls, visits, or anxious follow-ups, many offered a different perspective. Access to information through MyHealthNB often reduces their need to contact providers or visit clinics, streamlining routine tasks and minimizing unnecessary system use:

MyHealthNB should save the pharmacy and healthcare providers a lot of work. I recently looked up my COVID vaccination data because I am a caregiver for my mom, a lady with Alzheimer’s, and myself. We all go together to get our COVID shots. I would look up on MyHealthNB when our last one was, and it showed me I have to wait until the 22nd of April. It saves me calling the pharmacy, going to the pharmacy, asking the pharmacist about it. So it saves a huge thing on their shoulders.

Interviewee #1

### Overall Impact of MyHealthNB

Collectively, the qualitative findings suggest that MyHealthNB has the potential to empower citizens to take more proactive and informed roles in their health and health care. While complexity exists around interpreting new information and health system support constraints, most participants felt that the benefits outweighed the risks. When used in conjunction with supportive health care providers, MyHealthNB was perceived as a lever for improving patient preparedness, engagement, and health system efficiency. Multimedia Appendix 1 provides a detailed list of interviewee quotes, organized by specific impacts across the progression from enablement to empowerment, involvement, engagement, and cost-related outcomes.

### Quantitative Results

#### Overview

Building on the findings of the qualitative results, the quantitative results showed similar trends regarding the impact of MyHealthNB across enablement, empowerment, involvement, engagement, and cost-related outcomes.

#### Impacts of MyHealthNB on Enablement, Empowerment, Involvement, Engagement, and Costs

**Figure 3:**
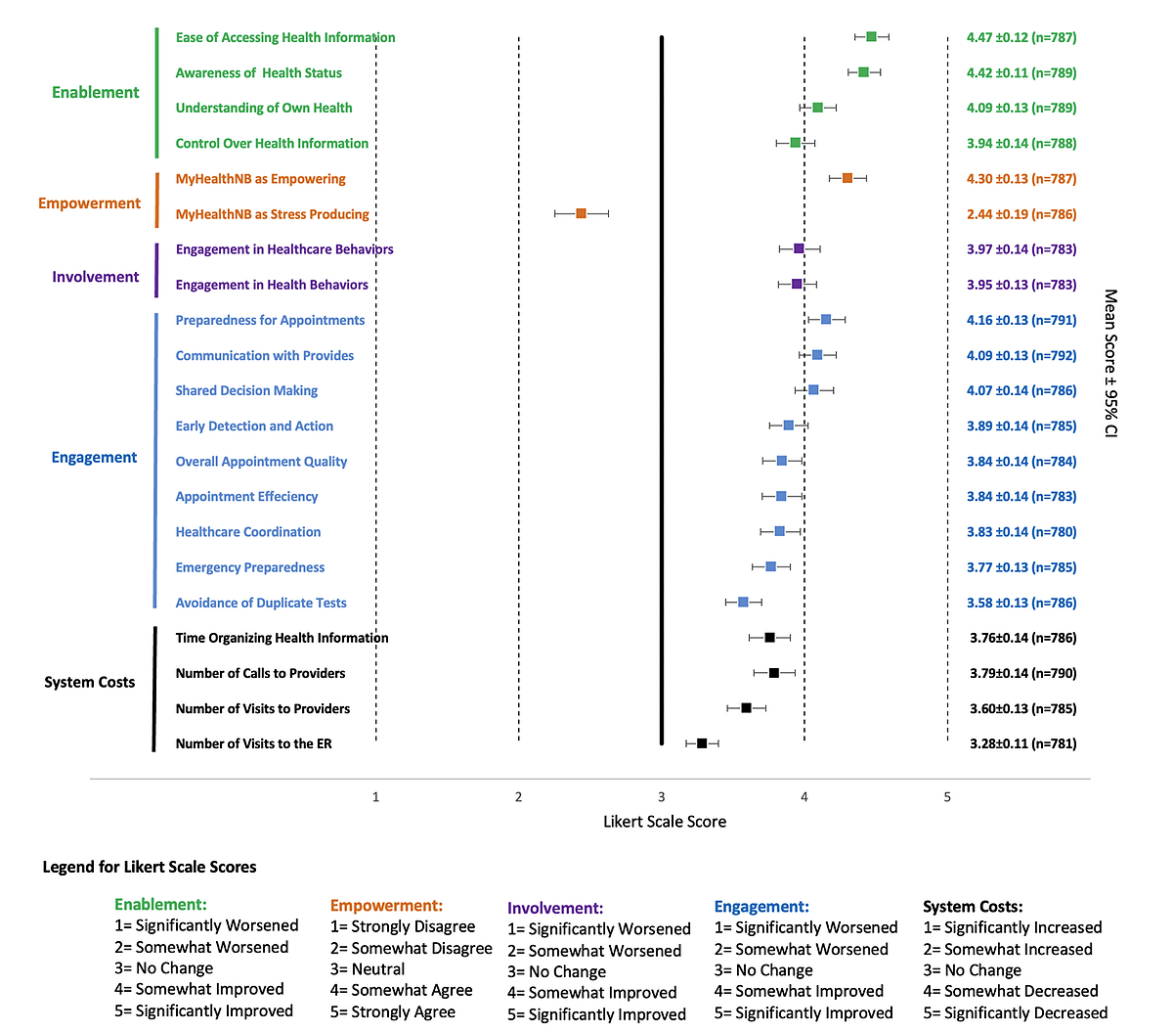
Self-reported impacts of MyHealthNB on 21 outcomes.

As illustrated in Figure 3, MyHealthNB was associated with statistically significant positive perceived impacts across all 5 domains of enablement, empowerment, involvement, engagement, and system costs, spanning all 21 measured outcomes (*P*<.05). The only outcome with a mean score below neutral was agreement that MyHealthNB was stress-producing, indicating that participants generally disagreed with this statement. A full description of the impact results, including descriptive statistics, is included in Multimedia Appendix 1.

#### Connection Between MyHealthNB Impacts: Mediation Analysis of Impact Pathways

**Figure 4:**
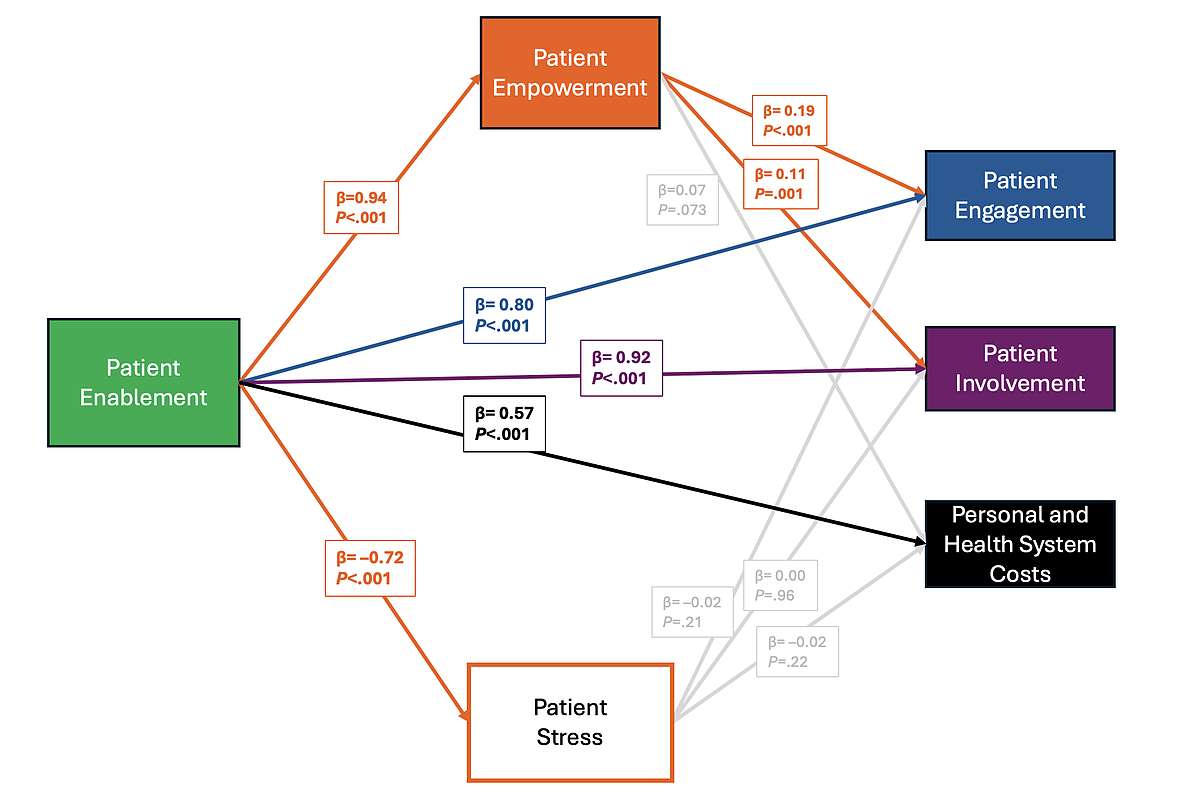
Mediation analysis of MyHealthNB impacts.

As shown in Figure 4, the results of the mediation analysis indicate patterns of association consistent with a hypothesized pathway where patients who feel more enabled by the PHR are also more likely to report feeling empowered, which in turn is associated with increased engagement and involvement in care.

Specifically, patients who felt more enabled by MyHealthNB were significantly more likely to feel empowered by it (*β*=0.94, *P*<.001), with enablement explaining over half of the variance in empowerment (*R*^2^=0.53). Empowerment, in turn, was associated with engagement and involvement in a manner consistent with partial mediation of the relationship between enablement and these behavioral outcomes. For engagement, enablement alone predicted outcomes (*β*=0.80, *P*<.001, *R*^2^=0.58), and both enablement (*β*=0.62, *P*<.001) and empowerment (*β*=0.19, *P*<.001) remained significant in the combined model, improving model fit (*R*^2^=0.61), consistent with partial mediation. For involvement, enablement alone was also predictive (*β*=0.92, *P*<.001, *R*^2^=0.54), and the addition of empowerment (*β*=0.11, *P*=.001) similarly improved model fit (*R*^2^=0.55), again consistent with partial mediation. In contrast, while enablement by MyHealthNB also predicted lower reported stress from MyHealthNB (*β*=–0.72, *P*<.001, *R*^2^=0.16), stress did not significantly predict patient engagement (*β*=–0.02, *P*=.21), patient involvement (*β*=0.00, *P*=.96), or cost-related outcomes (*β*=–0.02, *P*=.22), suggesting stress was not associated with outcomes in a manner consistent with a mediating role. For cost-related outcomes, enablement was independently predictive (*β*=0.57, *P*<.001, *R*^2^=0.29), but neither empowerment nor stress contributed explanatory value beyond enablement. Multimedia Appendix 1 includes the full mediation analysis results. All composite variables used in the mediation analyses demonstrated acceptable internal consistency. Enablement (4 items; Cronbach α=0.82), involvement (2 items; α=0.84; interitem *r*=0.73), engagement (9 items; α=0.96), and cost-related outcomes (4 items; α=0.81) supported aggregation of items into averaged composite scores. Empowerment and stress were each measured using single items; therefore, internal consistency reliability could not be assessed for these constructs.

#### Enabling Environments of MyHealthNB Impacts: Multiple Regression Analysis of Predictors

**Figure 5:**
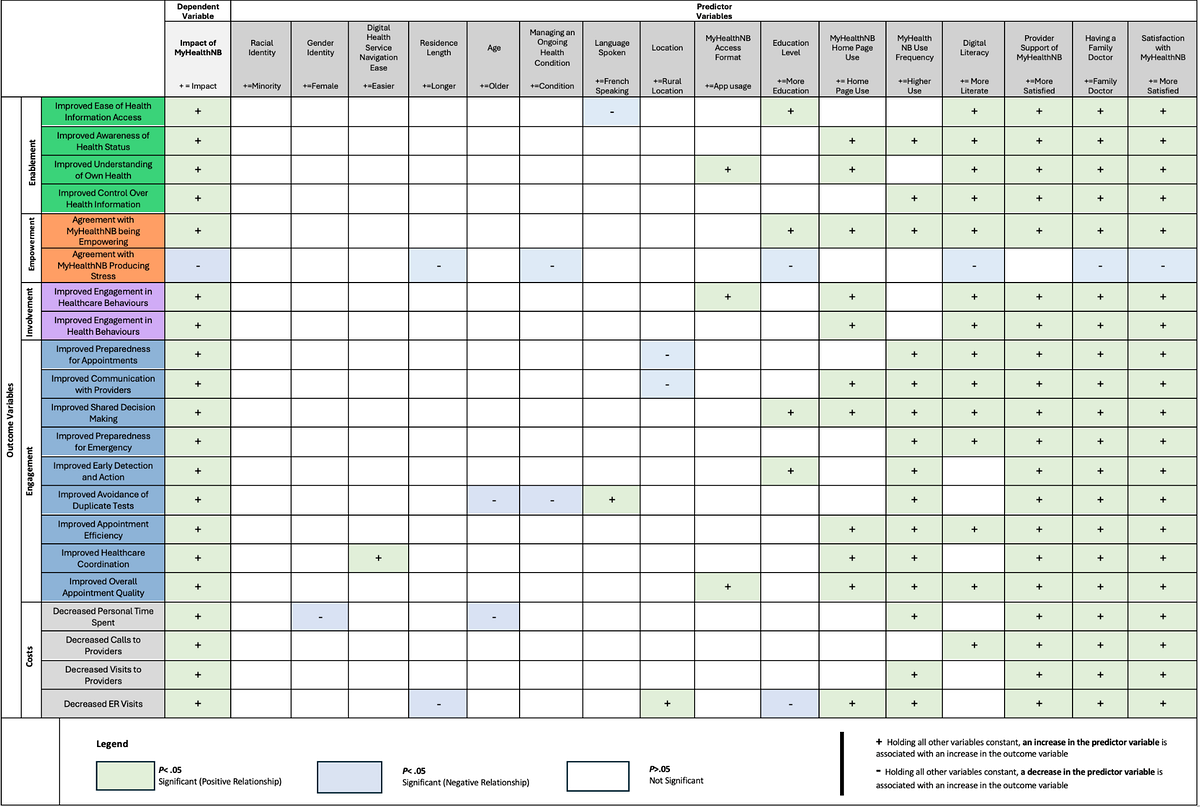
Map of MyHealthNB impacts and predictors.

Figure 5 presents the results of multivariable linear regression models used to identify factors that predict variations in self-reported impacts. A higher resolution image of Figure 5 can be found in the Multimedia Appendix 1. Satisfaction with MyHealthNB and having a family doctor were significantly associated with all 21 impacts (*P*<.05). In addition, provider support for MyHealthNB, digital literacy, and frequency of MyHealthNB use were significantly associated with 20, 15, and 15 impacts respectively (*P*<.05). Other variables, including use of the MyHealthNB homepage and education level were also significantly associated with specific impacts, although their associations were less consistent. Full details of these predictive factors, including effect sizes, significance levels, and multiple regression results, are provided in Multimedia Appendix 1**.** VIF values for the model examining predictors of MyHealthNB’s impact on ease of accessing health information ranged from 1.07 to 1.45, indicating no evidence of problematic multicollinearity (Multimedia Appendix 1 and Limitations section provide more detail).

## Discussion

### Summary of Findings

This mixed methods evaluation suggests that MyHealthNB, a province-wide PHR in New Brunswick, Canada, is associated with self-reported improvements in patient enablement, empowerment, involvement, engagement, and personal and health system costs. Qualitative findings revealed that MyHealthNB plays a foundational role in enabling patients through timely, accessible health information. This access fostered empowerment, with users describing greater peace of mind and control in managing their health. While some participants reported moments of increased stress when encountering unfamiliar or unexpected results, most described this stress as occurring alongside greater awareness and involvement in their care. Participants also reported perceived reductions in personal and health system costs, including fewer phone calls, appointments, and administrative burdens, suggesting that MyHealthNB may support more efficient use of both patient and system resources. Overall, participants saw MyHealthNB as a valuable way to promote deeper participation in their health and health care.

Quantitative findings aligned with qualitative insights, demonstrating MyHealthNB statistically significant improvements across 21 measures related to enablement, empowerment, involvement, engagement, and cost-related outcomes. Mediation analyses identified patterns of association consistent with a hypothesized pathway in which higher perceived enablement from MyHealthNB was associated with greater feelings of empowerment, alongside higher levels of patient involvement and engagement. Although greater enablement was also associated with lower reported stress related to MyHealthNB use, stress was not associated with involvement, engagement, or cost-related outcomes. Together, these findings suggest that empowerment may play a more central role than stress in shaping patient involvement and engagement, highlighting empowerment as a potentially important target for future intervention design and evaluation.

Multiple regression analysis identified key predictors of stronger impacts across all outcome domains, including satisfaction with MyHealthNB, having a family doctor, provider support for MyHealthNB, digital literacy, and MyHealthNB use frequency. These findings highlight the importance of both individual skills and broader system-level enablers to maximize the benefits of PHRs. Together, the qualitative and quantitative results indicate that interconnected, province-wide PHRs like MyHealthNB can strengthen empowerment-related outcomes.

### Key Implications

#### PHRs Can Influence Outcomes Related to Patient Empowerment and Behavior Change

This study speaks directly to calls for stronger empirical evidence linking PHR use to changes in patient empowerment and behavioral outcomes [10]. The quantitative and qualitative results suggest that province-wide PHRs like MyHealthNB are more than passive information repositories; they can be active drivers of patient empowerment and health behavior change, especially when use is supported by health care providers. Our findings echo insights from a large systematic review that assessed the broad impact of PHRs and found improvements in enablement (eg, increased knowledge), empowerment (eg, reassurance and reduced anxiety), involvement (eg, increased adherence to medications), and engagement (eg, improved consultations, patient-provider relationships) outcomes [36]. They also align with a conceptual synthesis and integrative review that examined literature supporting various PHR value propositions [35]. This synthesis identified evidence for PHRs improving value propositions around patient education, enablement, understanding, control, self-management, self-efficacy, preparation for emergencies, and provider communication [35]. This same synthesis also found evidence of reductions in personal and system-level costs, through savings in patient and provider time, reduced duplications of tests and treatments, and reduced burden on health care system resources [35]. Overall, our findings contribute to a growing body of evidence demonstrating PHRs’ impact on a range of patient and health system outcomes.

#### Complex Emotional Reactions Are Inherent to PHR Use

Our qualitative findings revealed that PHRs can simultaneously evoke empowerment and stress when using MyHealthNB. Importantly, these responses were not uniform across users, which led us to include follow-up survey questions examining both effects. Quantitative results showed that most respondents agreed MyHealthNB was empowering and disagreed that it led to stress; however, nearly one-quarter of respondents (23%) reported that using MyHealthNB was stress-inducing, indicating a meaningful minority of users experienced emotional burden.

The quantitative findings further suggested that higher perceived stress related to MyHealthNB use was associated with shorter length of residence in New Brunswick, not managing a chronic condition, lower education, lower digital health literacy, lack of a family doctor, and lower satisfaction with MyHealthNB as a platform. These patterns suggest that stress may arise when users have fewer experiential, informational, or clinical supports to help interpret and act on health information. For example, users with lower digital or health literacy and those newer to the health system may face greater uncertainty when reviewing results, while users without a regular primary care provider may lack opportunities to contextualize findings.

Our findings extend the literature on PHR-related stress [36,37], which has previously suggested that stress from access to health data might cause increased health care usage [38-40]. In our study, stress was not conceptualized as inherently beneficial or harmful, but rather as a natural response that may co-occur with increased awareness and engagement. Psychological research suggests that stress may represent a temporary and motivating form of heightened alertness when individuals feel more in control [41]. Moreover, uncertainty-reduction theory posits that access to reliable information can reduce overall anxiety by decreasing ambiguity, even if initial exposure to results is emotionally challenging [42]. These perspectives may help explain why many participants reported feeling empowered despite moments of stress. However, this interpretation remains speculative and warrants further investigation. Some participants described sustained anxiety or chose to disengage from MyHealthNB after encountering concerning results, underscoring the need for future research on long-term psychological impacts of PHR use.

Overall, the findings from this study underscore the importance of responsible PHR design and implementation that is sensitive to user context. Design strategies such as plain-language explanations, embedded educational resources, and clear guidance on next steps may be particularly valuable for users with fewer supports, helping ensure that greater access to information promotes empowerment rather than distress. Emerging applications of artificial intelligence (AI) may offer additional opportunities to support these goals [43-45]. For example, AI-generated plain-language summaries, contextual explanations of test results, and tailored guidance on urgency and next steps could help users better interpret their health information, particularly those with lower digital literacy or limited access to primary care [43-45]. Future research should evaluate whether such AI-enabled supports can enhance empowerment without compromising accuracy, transparency, and trust.

#### The Impact of PHRs Is Shaped by User Experience, Primary Care Access, and Provider Support

Our qualitative and quantitative results show that MyHealthNB’s impact was significantly shaped by key contextual factors: (1) satisfaction with MyHealthNB, (2) having a family doctor, (3) satisfaction with provider support for MyHealthNB, (4) digital literacy, and (5) MyHealthNB use frequency. In contrast, most demographic variables, such as gender, age, race, language, location of residence, length of residence, and managing an ongoing health condition, had small, inconsistent effects when controlling for other variables. Education level emerged as the most consistent demographic predictor. Higher education was associated with greater impact on self-reported ease of accessing health information, agreement with MyHealthNB being empowering, engagement in shared decision-making, and early detection and action. Conversely, lower education was associated with higher reports of stress from using MyHealthNB. This pattern aligns with prior research showing that individuals with lower education levels are more likely to experience heightened health-related anxiety [46,47].

Among usage-related variables, MyHealthNB home page use was positively associated with most outcomes. This suggests that deeper use of MyHealthNB (eg, accessing more features and more interactions with personal and health systems information) contributed to greater perceived impact across enablement, empowerment, involvement, engagement, and cost-related domains. Satisfaction with MyHealthNB, digital literacy, and MyHealthNB use frequency were among the most consistent and expected predictors of impact, reinforcing prior research showing that ease of use, digital competence, and system familiarity are key factors influencing behavior change through digital health tools [48].

Having a family doctor and feeling supported by a provider were strongly associated with users reporting that MyHealthNB helped them feel more enabled, empowered, involved, engaged, and efficient in managing their care. This is especially relevant in New Brunswick, Canada, where access to primary care remains a challenge [49], and where 23% of survey respondents reported not having a family doctor. Many of the benefits attributed to MyHealthNB, including improved understanding of health information, better preparation for appointments, and more effective communication with providers, may depend on an ongoing supportive relationship with a provider who can help interpret and act on information. Our results reinforce the importance of embedding digital health tools within supportive care environments [50-52]. Even well-designed PHRs may be contingent on the strength of the health care ecosystem in which they are used. Without adequate access to primary care and active provider engagement, PHRs risk becoming isolated tools rather than integrated supports that improve patient care and system efficiency. PHR implementers should prioritize strategies that strengthen provider engagement and meaningfully integrate PHRs into primary care workflows.

### Strengths and Limitations

This study is the first academic mixed methods evaluation of a province-wide PHR in Canada, exploring impacts on patient empowerment-related outcomes. A major strength of this study is its pragmatic, developmental mixed methods design, which enabled iterative refinement of research priorities, alignment with real-world implementations, and integration of qualitative insights into the quantitative survey. The large and relatively representative samples in both phases supported robust analyses, including mediation and regression modeling. Overall, the study demonstrated how a PHR like MyHealthNB can support patient-reported improvements across empowerment-related domains, while also identifying contextual factors that influence its impact.

This study has several limitations. Recruitment was conducted exclusively online (via email and web), which may have limited participation to individuals with internet access and higher levels of digital engagement. Measures related to internet or device access barriers were not included. Participation was voluntary and self-selected, and males and individuals with lower education levels were underrepresented, potentially biasing the sample towards an overestimation of perceived benefits. All data were self-reported, introducing potential for recall bias and social desirability bias. Although we aimed to use neutral language when asking participants to reflect on changes related to MyHealthNB, these responses, particularly those regarding reduced provider calls and visits, should be interpreted with caution. Future research could incorporate objective usage data to validate self-reported impacts. The cross-sectional survey design was appropriate for the study’s pragmatic goals, but it limits the ability to draw causal conclusions.

Additionally, while survey items were informed by qualitative findings, prior Canadian surveys, codevelopment with stakeholders, and relevant literature, they were not drawn from validated scales. Many constructs were assessed using single-item measures, which precluded assessment of internal consistency and increased susceptibility to measurement error and response bias, therefore limiting reliability and construct validity. As a result, observed associations should be interpreted as patterns of perceived impact rather than precise estimates of underlying constructs. The findings in this study should be considered exploratory and hypothesis-generating, pending newly developed measures.

Several limitations specific to the mediation analyses should also be noted. Although mediation models were used to examine theoretical impact pathways, the cross-sectional nature of the survey data precludes confirmation of temporal ordering among enablement, empowerment, stress, and downstream behavioral outcomes. As a result, these analyses can only identify associations consistent with hypothesized pathways rather than demonstrate causal mediating processes or directional mechanisms. Formal indirect effects were not estimated, as doing so would risk overstating causal inference in the absence of longitudinal or experimental data. Accordingly, mediation findings should be interpreted as theory-consistent and exploratory, intended to inform future hypothesis testing rather than confirm causal pathways.

Multicollinearity was only formally assessed for the regression model examining MyHealthNB’s impact on ease of accessing health information. Although the same predictors were used in all models and similar multicollinearity patterns may be expected, VIFs were not recalculated separately for each model and may vary across analyses due to differences in missing data.

Findings from this study should be interpreted within the broader health system context of New Brunswick, which is characterized by primary care shortages, variable digital literacy, and a PHR that primarily provides access to information rather than fully integrated provider-facing functions (eg, instant messaging). In larger provinces or jurisdictions with more mature digital infrastructure and primary care access, the impacts observed here may be amplified or moderated by existing supports and workflows. Evaluating other Canadian PHRs may suggest that provider messaging, linked educational resources, and clearer clinical follow-up pathways may play an important role in shaping perceived impact. Overall, PHR impacts are not solely a function of the technology itself, but of the surrounding health system context, implementation strategy, and availability of supports.

Finally, this paper reports on one component of a broader evaluation examining the design, implementation, and impacts of MyHealthNB in New Brunswick. Future publications will present additional findings, including barriers and facilitators to design and implementation, and perspectives from individuals who do not use MyHealthNB.

### Conclusions

This study used an exploratory sequential mixed methods approach to examine how MyHealthNB, a province-wide PHR in New Brunswick, Canada, impacted patient empowerment-related outcomes. Our findings show that MyHealthNB users see MyHealthNB as more than a way to access information, recognizing it as something that actively supports enablement, empowerment, involvement, engagement, and cost-related improvements. The most impactful experiences with MyHealthNB occurred when patients had access to a family doctor and received support from providers, highlighting the importance of embedding digital tools within trusted care relationships. Although some users experienced stress when interacting with MyHealthNB, this did not diminish its overall benefits. Overall, citizens report broad benefits from using a PHR, but realizing its full impact depends on user confidence with the digital experience and strong integration with primary care.

## Appendix

Multimedia Appendix 1 Additional study details.

## Abbreviations

AI: artificial intelligence
PHR: personal health record
VIF: variance inflation factor

## Data Availability

Data generated or analyzed during this study are not publicly available, as participants did not consent to share the data outside of the research team.
