# Supplementary material for: Exploring Patient Empowerment and Health System Outcomes Associated With MyHealthNB, a Provincial Personal Health Record System: Exploratory Mixed Methods Study
Source: J Med Internet Res. 2026 Apr 28;28:e87521. doi: 10.2196/87521 (PMC13168860; doi:10.2196/87521)
Supplement: Multimedia Appendix 1 [file jmir_v28i1e87521_app1.docx]

# Multimedia Appendix

## Part 1: Details about MyHealthNB

**Additional Information about the features of MyHealthNB at the time of the study:**

- Interviews were April 2024 to August 2024
- Survey was November 27th to January 11th, 2025

**MyHealthNB Access:**

**At the time of this study, MyHealthNB was available via:**

- Mobile app
- Web portal

**Access to personal health records was only available to:**

- Those over the age of 16, with NB Medicare
- Those who previously had full access during COVID-19 via obtaining a registration code at a vaccination clinic or assessment center
- Those who obtained a registration code via Service New Brunswick Service Center or while getting bloodwork. In late 2024 registration codes could also be obtained during x-rays or other appointments at Horizon or Vitalité Healthcare facilities.

**MyHealthNB Content:**

MyHealthNB aggregates selected health information from provincial data sources. Specifically, laboratory results and medical imaging reports are drawn from New Brunswick’s two regional health authorities (Vitalité Health Network and Horizon Health Network). Vaccination records include most publicly funded vaccines captured in the provincial Public Health Information Solution (PHIS), regardless of whether vaccines were administered by public health, family physicians, nurses, or pharmacists. Medication information is sourced from community pharmacies, and visit history reflects hospital encounters within the two regional health authorities. Services delivered outside these systems, such as privately funded tests or care provided outside New Brunswick, are not comprehensively captured.

At the time of this study, MyHealthNB users had access to:

**A “Home” page that included:**

- Guidance on accessing healthcare (e.g., how-to links for finding services)
- Health System Data (e.g., wait time information)
- Information on social supports (e.g., programs and services)
- Self-scheduling tools (e.g., x-ray booking and eVisitNB)
- General health system resources (e.g., links to mental health support, regional health authority websites)
- Link to MyHealthRecords (see below)

**A “MyHealth Records” application that included:**

- Lab Results
  - Data is provided through the provincial Electronic Health Record (EHR). Only finalized results are displayed. There is a 14-day delay on sensitive results once finalized to provide time for health-care providers to review and support next steps. Microbiology, cytopathology, pathology, transfusion medicine, and molecular genetics results are accessible dating back to June 1, 2023. All other lab results are accessible as far back as 2010. Data is displayed in the language of its originator, and may appear in English, French or both.
- Immunization records
  - Displays all immunization records stored in the Public Health Information Solution (PHIS). The vaccines listed may not include all immunizations received.
- Medications
  - Includes medications filled and refilled by community pharmacies in the province from the Drug Information System (DIS). Not included: Prescriptions not yet filled by a pharmacy, samples given by healthcare providers, medications given by hospitals or clinics.
- Imaging Reports
  - Data is provided through the provincial Electronic Health Record (EHR). Only finalized reports are displayed. There is a 14-day delay on all reports once finalized, to provide time for health-care providers to review and support next steps. Imaging reports are accessible dating back to June 1, 2023.

**A “Patient Mediated Patient Summary” application:**

- Available in Mobile app – July 2024
- Available in Web portal – December 2024
- It is a standardized summary of a patient’s health information in MyHealth Records that is viewable for 24 hours and includes approximately 12 months of latest medications, immunizations, lab, and medical imaging tests.

**A “COVID-19 Application” that included:**

- Available in Web portal only
- COVID-19 vaccinations, Canadian Proof of Vaccination (QR Code credential), COVID-19 PCR test results
- Ability for parents to add dependent children under 16, with NB Medicare and view their COVID-19 vaccinations and test results and generate the Canadian Proof of Vaccination.

Of note, at the time of this study, MyHealthNB did not include: secure messaging between patients and providers, the ability for patients to upload their own health information, and access to individuals under the age of 16.


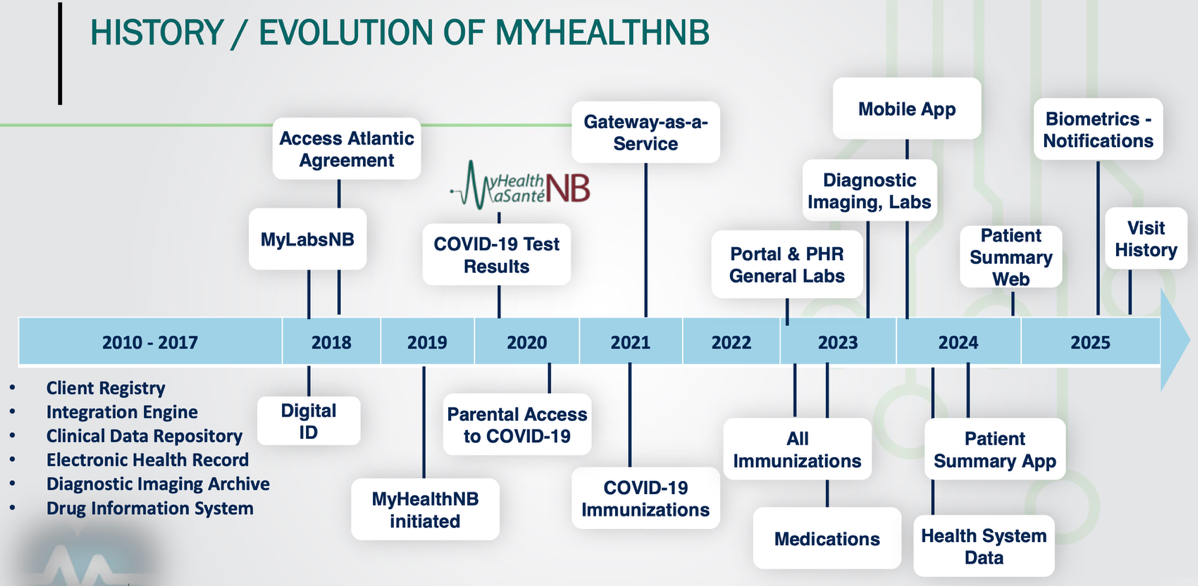


## Part 2: Details about MyHealthNB Survey Measures

**Dependent Variables (i.e. MyHealthNB Impacts)**

| **Domain** | **Question Prompt and Scale** | **Measures** |
| --- | --- | --- |
| **Patient Enablement** | Prompt of *“How has having access to your personal health records through MyHealthNB impacted the following?”* with a 5-Point Likert Scale including, *“Significantly Improved (5)”, “Somewhat Improved (4)”, “No Change (3)”, “Somewhat Worsened (2)”,* and *“Significantly Worsened (1)”* | 1. **Ease in accessing your health information** (e.g., ability to find a specific health record) 2. **Awareness of your health status** (e.g., awareness of your results, vaccination status, or medication history) 3. **Understanding of your health** (e.g., understanding why you are experiencing a certain issue or why you need a specific treatment) 4. **Control over your health information** (e.g., ability to identify errors in your record or share your health information with who you want to) |
| **Patient Empowerment** | Prompt of *“Rate your level of agreement with the following statement”* with a 5-Point Likert Scale including, *“Strongly agree (5)”, “Somewhat agree (4)”, “Neither agree nor disagree (3)”, “Somewhat disagree (2)”,* and *“Strongly disagree (1)”* | 1. **"MyHealthNB has empowered me to take a more active role in my health and healthcare** by giving me greater access, control, and understanding of my health information." 2. "**MyHealthNB has raised concerns, frustration, and stress** for me because of the ongoing challenges I face in accessing, managing, and understanding my health information." |
| **Patient Involvement** | Prompt of *“How has having access to your personal health records through MyHealthNB impacted the following?”* with a 5-Point Likert Scale including, *“Significantly Improved (5)”, “Somewhat Improved (4)”, “No Change (3)”, “Somewhat Worsened (2)”,* and *“Significantly Worsened (1)”* | 1. **Engagement in your healthcare** (e.g., doing preventative screenings, scheduling follow up appointments, refilling prescriptions, managing an ongoing issue, etc.) 2. **Engagement in your health** (e.g., making healthier decisions, setting goals related to your health, etc.) |
| **Patient Engagement** | Prompt of *“How has having access to your personal health records through MyHealthNB impacted the following?”* with a 5-Point Likert Scale including, *“Significantly Improved (5)”, “Somewhat Improved (4)”, “No Change (3)”, “Somewhat Worsened (2)”,* and *“Significantly Worsened (1)”* | 1. **Preparedness for visits with a healthcare provider** (e.g., having a plan for a concern, result, or treatment you want to discuss) 2. **Communication with a healthcare provider** (e.g., being able to discuss a concern, result, or treatment with a provider) 3. **Ability to be a more active participant in healthcare decision-making** (e.g., collaborating with a provider to choose the best treatment or next step) 4. **Preparedness for a health emergency or unexpected health event** (e.g., ability to quickly access what you need to share during an emergency) 5. **Early detection and action** (e.g., more timely seeking and addressing an abnormality) 6. **Ability to avoid taking a test or treatment you don't need or want** (e.g., avoiding a duplicate test or treatment) 7. **Efficiency of your healthcare appointments** (e.g., having more time to talk about what matters to you or getting treatment faster because you know your medical history) 8. **Coordination of your healthcare** (e.g., ensuring all of your healthcare providers are on the same page) 9. **Overall quality of your healthcare appointments** (e.g., satisfaction with your overall experience with visiting a provider) |
| **Personal and Health System Cost** | Prompt of *“How has having access to your personal health records through MyHealthNB impacted the following?”* with a 5-Point Likert Scale including, *“Significantly Decreased (5)”, “Somewhat Decreased (4)”, “No Change (3)”, “Somewhat Increased (2)”,* and *“Significantly Increased (1)”* | 1. **Time spent finding and organizing your health information** (e.g., time spent retrieving your health results from different providers or clinics) 2. **Number of calls you have to make to a healthcare provider to get health information** (e.g., information about test results, vaccine records, medication information, treatment history, etc.) 3. **Number of times you travel to a healthcare provider to get health information** (e.g., travelling to receive a result) 4. **Number of times you visit the emergency room** |

**Independent Variables (i.e., Predictors of MyHealthNB Impacts)**

| **Domain** | **Scale** | **Measures** |
| --- | --- | --- |
| **Age** | Multiple choice scale asking, “What is your age?” | 1. 19-24 2. 25-34 3. 35-44 4. 45-54 5. 55-64 6. 65-74 7. 75-84 8. 85+ 9. Prefer not to say |
| **Race** | Select all that apply asking, “What is your racial identity?” | 1. White (e.g., Caucasian, European Decent) 2. Indigenous (e.g., First Nations, Inuit, Métis) 3. Black (e.g., African-Canadian, African, Afro-Caribbean descent) 4. Latino (e.g., Latin American, Hispanic descent) 5. Middle Eastern (e.g., Arab, Persian, West Asian descent, e.g. Afghan, Egyptian, Iranian, etc.) 6. South Asian (e.g. East Indian, Pakistani, Sri Lankan, Indo-Caribbean, other South Asian descent etc.) 7. East Asian (e.g., Chinese, Korean, Japanese, Taiwanese descent) (4) 8. Southeast Asian (e.g., Filipino, Vietnamese, Cambodian, Thai, other Southeast Asian descent) 9. Prefer not to say 10. Prefer to self-identify: |
| **Gender** | Multiple choice asking, “What is your gender identity?” | 1. Male 2. Female 3. Non-binary 4. Prefer not to say 5. Prefer to self-identify: |
| **Location*** | Open text | 1. Which area of New Brunswick do you currently reside in? [Specify a city, town, or region] |
| **Language** | Multiple choice asking, “Which of Canada's official languages are you most comfortable with?” | 1. Both English and French 2. English 3. French 4. Neither English nor French 5. Prefer not to say 6. Other |
| **Residence Length** | Multiple choice asking, “How long have you been residing in New Brunswick?” | 1. 0-3 years 2. 3-10 years 3. 10-20 years 4. 20+ years 5. Prefer not to say |
| **Education Level** | Multiple choice asking, “What is the highest level of education you have completed?” | 1. Some elementary school 2. Completed elementary school 3. Some high school 4. Completed high school 5. Some post-secondary education (college, university, trade/vocational) 6. Completed college diploma or certificate 7. Completed university degree (Bachelor's) 8. Some graduate or professional studies 9. Completed graduate or professional degree (Master's, Ph.D., M.D., etc.) 10. Prefer not to say |
| **Web Confidence** | Multiple choice asking, “How confident do you feel navigating websites on the internet?” | 1. Very confident 2. Somewhat confident 3. Not very confident 4. Not confident at all 5. I don't use the internet |
| **Digital Health Navigation** | Multiple choice asking, " How would you describe your experience navigating the different digital health services available in New Brunswick?” | 1. I find it very challenging to navigate 2. I find it somewhat challenging to navigate 3. I do not find it very challenging to navigate 4. I do not find it challenging at all to navigate 5. I have not attempted to navigate any digital health services in New Brunswick 6. Other |
| **Ongoing Health Condition** | Multiple choice asking, “Are you currently managing an ongoing health issue?” | 1. Yes 2. No 3. Other |
| **Family Doctor Status** | Multiple choice asking, “Do you currently have a family doctor?” | 1. Yes 2. No 3. Other |
| **MyHealthNB Access Format** | Select all that apply asking, “How have you accessed MyHealthNB?” | 1. MyHealthNB web portal (i.e., via the internet) 2. MyHealthNB app (i.e., downloaded in the App Store) |
| **MyHealthNB Satisfaction Score** | Multiple choice asking, “How satisfied are you with the MyHealthNB web portal?” and “How satisfied are you with the MyHealthNB app?” | 1. I am very satisfied 2. I am somewhat satisfied 3. I am somewhat dissatisfied 4. I am very dissatisfied |
| **MyHealthNB Usage Frequency** | Multiple choice asking, “How often do you typically log into MyHealthNB” | 1. Multiple times per day 2. Daily 3. Weekly 4. Monthly 5. Occasionally 6. Rarely |
| **MyHealthNB Home Page Use** | Select all that apply asking, “What general health information have you accessed from the ‘Home’ page in the MyHealthNB mobile app or under ‘Important Health Resources’ on the website?” | 1. Health Systems Data (e.g., wait times information) 2. Accessing Healthcare (e.g., information about how to access care) 3. Social Supports NB (e.g., social programs and services) 4. Self-Scheduling (e.g., x-ray booking or eVisitNB) 5. General Resources (e.g., links to Horizon or Vitalité pages, 211, NB Health Link) 6. Bridge the Gap 7. Respiratory Watch 8. None; I have not accessed any additional health information on MyHealthNB 9. Other |
| **MyHealthNB Provider Support** | Multiple choice asking, “How satisfied do you feel with the level of encouragement your healthcare providers have shown towards your use of MyHealthNB?” | 1. I feel very satisfied with the level of encouragement 2. I feel somewhat satisfied with the level of encouragement 3. I feel neither satisfied nor dissatisfied about the level of encouragement 4. I feel somewhat dissatisfied about the level of encouragement 5. I feel very dissatisfied about the level of encouragement |

*Please note, location open text data was recoded into New Brunswick’s health zones listed here: <https://nbhc.ca/data/browse/health-zones>

## Part 3: Impact of MyHealthNB – Quantitative and Qualitative Results

**Higher Resolution of Figure 5 (Below)**

|  | | | | **Dependent Variable** | **Predictor**  **Variables** | | | | | | | | | | | | | | | |
| --- | --- | --- | --- | --- | --- | --- | --- | --- | --- | --- | --- | --- | --- | --- | --- | --- | --- | --- | --- | --- |
|  |  |  |  | **Impact of MyHealthNB** | Racial Identity | Gender Identity | Digital Health Service Navigation Ease | Residence Length | Age | Managing an Ongoing Health Condition | Language Spoken | Location | MyHealthNB Access Format | Education Level | MyHealthNB Home Page Use | MyHealthNB Use Frequency | Digital Literacy | Provider Support of MyHealthNB | Having a Family Doctor | Satisfaction with MyHealthNB |
|  |  |  |  | + = Impact | +=Minority | +=Female | +=Easier | +=Longer | +=Older | +=Condition | +=French Speaking | +=Rural Location | +=App usage | +=More Education | += Home Page Use | +=Higher Use | += More Literate | +=More Satisfied | +=Family Doctor | += More Satisfied |
| **Outcome Variables** | **Enablement** | Improved Ease of Health Information Access | | + |  |  |  |  |  |  | – |  |  | + |  |  | + | + | + | + |
|  |  | Improved Awareness of Health Status | | + |  |  |  |  |  |  |  |  |  |  | + | + | + | + | + | + |
|  |  | Improved Understanding of Own Health | | + |  |  |  |  |  |  |  |  | + |  | + |  | + | + | + | + |
|  |  | Improved Control Over Health Information | | + |  |  |  |  |  |  |  |  |  |  |  | + | + | + | + | + |
|  | **Empowerment** | Agreement with MyHealthNB being Empowering | | + |  |  |  |  |  |  |  |  |  | + | + | + | + | + | + | + |
|  |  | Agreement with MyHealthNB Producing Stress | | – |  |  |  | – |  | – |  |  |  | – |  |  | – |  | – | – |
|  | **Involvement** | Improved Engagement in Healthcare Behaviours | | + |  |  |  |  |  |  |  |  | + |  | + |  | + | + | + | + |
|  |  | Improved Engagement in Health Behaviours | | + |  |  |  |  |  |  |  |  |  |  | + |  | + | + | + | + |
|  | **Engagement** | Improved Preparedness for Appointments | | + |  |  |  |  |  |  |  | – |  |  |  | + | + | + | + | + |
|  |  | Improved Communication with Providers | | + |  |  |  |  |  |  |  | – |  |  | + | + | + | + | + | + |
|  |  | Improved Shared Decision Making | | + |  |  |  |  |  |  |  |  |  | + | + | + | + | + | + | + |
|  |  | Improved Preparedness for Emergency | | + |  |  |  |  |  |  |  |  |  |  |  | + | + | + | + | + |
|  |  | Improved Early Detection and Action | | + |  |  |  |  |  |  |  |  |  | + |  | + |  | + | + | + |
|  |  | Improved Avoidance of Duplicate Tests | | + |  |  |  |  | – | – | + |  |  |  |  | + |  | + | + | + |
|  |  | Improved Appointment Efficiency | | + |  |  |  |  |  |  |  |  |  |  | + | + | + | + | + | + |
|  |  | Improved Healthcare Coordination | | + |  |  | + |  |  |  |  |  |  |  | + | + |  | + | + | + |
|  |  | Improved Overall Appointment Quality | | + |  |  |  |  |  |  |  |  | + |  | + | + | + | + | + | + |
|  | **Costs** | Decreased Personal Time Spent | | + |  | – |  |  | – |  |  |  |  |  |  | + |  | + | + | + |
|  |  | Decreased Calls to Providers | | + |  |  |  |  |  |  |  |  |  |  |  |  | + | + | + | + |
|  |  | Decreased Visits to Providers | | + |  |  |  |  |  |  |  |  |  |  |  | + |  | + | + | + |
|  |  | Decreased ER Visits | | + |  |  |  | – |  |  |  | + |  | – | + | + |  | + | + | + |
|  | | | **P< .05** Positive Relationship  ***P*>.05** Not Significant  **P< .05** Negative Relationship  **+**  Holding all other variables constant, **an increase in the predictor variable** is associated with an increase in the outcome variable  – Holding all other variables constant, **a decrease in the predictor variable** is associated with an increase in the outcome variable  **Legend** | | | | | | | | | | | | | | | | | |

### A. Impact of MyHealthNB on Enablement Outcomes

**Table 1.** Impacts of MyHealthNB on Enablement: T-Test Results

| **Outcome** | **Mean** | **SD** | **95% CI** | **n** | **p-value** |
| --- | --- | --- | --- | --- | --- |
| Ease of Access | 4.47 | 0.83 | 4.41–4.53 | 787 | <0.001 |
| Awareness of Health Status | 4.42 | 0.82 | 4.36–4.48 | 789 | <0.001 |
| Understanding of Health | 4.09 | 0.92 | 4.03–4.16 | 789 | <0.001 |
| Control Over Health Info | 3.94 | 0.97 | 3.87–4.01 | 788 | <0.001 |

**Table 2**. Impacts of MyHealthNB on Enablement: Descriptive Statistics of Likert Scores

| **Outcome** | **5 (Significantly Improved)** | **4 (Somewhat Improved)** | **3**  **(No Change)** | **2 (Somewhat Worsened)** | **1 (Significantly Worsened)** | **Total** |
| --- | --- | --- | --- | --- | --- | --- |
| Ease of Access | 492 (62.5%) | 211 (26.8%) | 62 (7.9%) | 7 (0.9%) | 15 (1.9%) | 787 |
| Awareness of Health Status | 457 (57.9%) | 234 (29.7%) | 80 (10.1%) | 7 (0.9%) | 11 (1.4%) | 789 |
| Understanding of Health | 322 (40.8%) | 256 (32.5%) | 186 (23.6%) | 13 (1.6%) | 12 (1.5%) | 789 |
| Control Over Health Info | 284 (36.0%) | 218 (27.7%) | 255 (32.4%) | 14 (1.8%) | 17 (2.2%) | 788 |

**Figure 1.** Impacts of MyHealthNB on Enablement: Descriptive Statistics of Likert Scores

### B. Impact of MyHealthNB on Empowerment Outcomes

**Table 1.** Impacts of MyHealthNB on Empowerment: T-Test Results

| **Outcome** | **Mean** | **SD** | **95% CI** | **n** | **p-value** |
| --- | --- | --- | --- | --- | --- |
| Agree MyHealthNB is Empowering | 4.3 | 0.93 | 4.24–4.37 | 787 | <.001 |
| Agree MyHealthNB is Stressing | 2.44 | 1.34 | 2.35–2.53 | 786 | <.001 |

**Table 2**. Impacts of MyHealthNB on Empowerment: Descriptive Statistics of Likert Scores

| **Outcome** | **5 (Strongly Agree)** | **4 (Somewhat Agree)** | **3 (Neutral)** | **2 (Somewhat Disagree)** | **1 (Strongly Disagree)** | **Total** |
| --- | --- | --- | --- | --- | --- | --- |
| Agree MyHealthNB is Empowering | 421 (53.5%) | 240 (30.5%) | 89 (11.3%) | 18 (2.3%) | 19 (2.4%) | 787 |
| Agree MyHealthNB is Stressing | 78 (9.9%) | 102 (13.0%) | 179 (22.8%) | 155 (19.7%) | 272 (34.6%) | 786 |

**Figure 1.** Impacts of MyHealthNB on Empowerment: Descriptive Statistics of Likert Scores

### C. Impact of MyHealthNB on Involvement Outcomes

**Table 1.** Impacts of MyHealthNB on Involvement: T-Test Results

| **Outcome** | **Mean** | **SD** | **95% CI** | **n** | **p-value** |
| --- | --- | --- | --- | --- | --- |
| Engagement in Healthcare | 3.97 | 1 | 3.90–4.04 | 783 | <.001 |
| Engagement in Health | 3.95 | 0.94 | 3.89–4.01 | 783 | <.001 |

**Table 2**. Impacts of MyHealthNB on Involvement: Descriptive Statistics of Likert Scores

| **Outcome** | **5 (Significantly Improved)** | **4 (Somewhat Improved)** | **3**  **(No Change)** | **2 (Somewhat Worsened)** | **1 (Significantly Worsened)** | **Total** |
| --- | --- | --- | --- | --- | --- | --- |
| Engagement in Health Behaviors | 283 (36.1%) | 209 (26.7%) | 271 (34.6%) | 7 (0.9%) | 13 (1.7%) | 783 |
| Engagement in Healthcare Behaviors | 296 (37.8%) | 223 (28.5%) | 228 (29.1%) | 13 (1.7%) | 23 (2.9%) | 783 |

**Figure 1.** Impacts of MyHealthNB on Involvement: Descriptive Statistics of Likert Scores

### D. Impact of MyHealthNB on Engagement Outcomes

**Table 1.** Impacts of MyHealthNB on Engagement: T-Test Results

| **Outcome** | **Mean** | **SD** | **95% CI** | **n** | **p-value** |
| --- | --- | --- | --- | --- | --- |
| Appointment Preparedness | 4.16 | 0.9 | 4.09–4.22 | 791 | <.001 |
| Communication with Provider | 4.09 | 0.94 | 4.03–4.16 | 792 | <.001 |
| Decision Making | 4.07 | 0.97 | 4.00–4.14 | 786 | <.001 |
| Early Detection | 3.89 | 0.97 | 3.82–3.96 | 785 | <.001 |
| Quality of Appointments | 3.84 | 0.96 | 3.78–3.91 | 784 | <.001 |
| Efficiency | 3.84 | 0.98 | 3.77–3.91 | 783 | <.001 |
| Coordination of Care | 3.83 | 0.98 | 3.76–3.90 | 780 | <.001 |
| Emergency Preparedness | 3.77 | 0.95 | 3.70–3.83 | 785 | <.001 |
| Duplicate Avoidance | 3.58 | 0.9 | 3.51–3.64 | 786 | <.001 |

**Table 2**. Impacts of MyHealthNB on Engagement: Descriptive Statistics of Likert Scores

| **Outcome** | **5 (Significantly Improved)** | **4 (Somewhat Improved)** | **3**  **(No Change)** | **2 (Somewhat Worsened)** | **1 (Significantly Worsened)** | **Total** |
| --- | --- | --- | --- | --- | --- | --- |
| Preparedness for Appointments | 355 (44.9%) | 232 (29.3%) | 187 (23.6%) | 6 (0.8%) | 11 (1.4%) | 791 |
| Communication with Providers | 335 (42.3%) | 237 (29.9%) | 191 (24.1%) | 16 (2.0%) | 13 (1.6%) | 792 |
| Shared Decision Making | 334 (42.5%) | 215 (27.4%) | 210 (26.7%) | 10 (1.3%) | 17 (2.2%) | 786 |
| Emergency Preparedness | 222 (28.3%) | 202 (25.7%) | 333 (42.4%) | 13 (1.7%) | 15 (1.9%) | 785 |
| Early Detection and Action | 269 (34.3%) | 207 (26.4%) | 279 (35.5%) | 14 (1.8%) | 16 (2.0%) | 785 |
| Avoidance of Duplicate Tests | 172 (21.9%) | 147 (18.7%) | 441 (56.1%) | 13 (1.7%) | 13 (1.7%) | 786 |
| Healthcare Coordination | 251 (32.2%) | 197 (25.3%) | 299 (38.3%) | 15 (1.9%) | 18 (2.3%) | 780 |
| Efficiency of Appointments | 251 (32.1%) | 209 (26.7%) | 292 (37.3%) | 10 (1.3%) | 21 (2.7%) | 783 |
| Overall Quality of Appointments | 242 (30.9%) | 232 (29.6%) | 273 (34.8%) | 20 (2.6%) | 17 (2.2%) | 784 |

**Figure 1.** Impacts of MyHealthNB on Engagement: Descriptive Statistics of Likert Scores

### E. Impact of MyHealthNB on System Cost Outcomes

**Table 1.** Impacts of MyHealthNB on Costs: T-Test Results

| **Outcome** | **Mean** | **SD** | **95% CI** | **n** | **p-value** |
| --- | --- | --- | --- | --- | --- |
| Cost - Time | 3.76 | 1.04 | 3.69–3.83 | 786 | <.001 |
| Cost - Calls | 3.79 | 1.06 | 3.72–3.86 | 790 | <.001 |
| Cost - Visits | 3.59 | 0.92 | 3.53–3.66 | 785 | <.001 |
| Cost - ER | 3.28 | 0.66 | 3.23–3.34 | 781 | <.001 |

**Table 2**. Impacts of MyHealthNB on Costs: Descriptive Statistics of Likert Scores

| **Outcome** | **5**  **(Significantly Decreased)** | **4**  **(Somewhat Decreased)** | **3**  **(No Change)** | **2**  **(Somewhat Increased)** | **1**  **(Somewhat Increased)** | **Total** |
| --- | --- | --- | --- | --- | --- | --- |
| Time Spent Organizing Info | 231 (29.4%) | 218 (27.7%) | 273 (34.8%) | 43 (5.5%) | 21 (2.7%) | 786 |
| Calls to Providers | 251 (31.8%) | 202 (25.6%) | 278 (35.2%) | 37 (4.7%) | 22 (2.8%) | 790 |
| Provider Visits | 179 (22.8%) | 178 (22.7%) | 378 (48.2%) | 31 (4.0%) | 19 (2.4%) | 785 |
| ER Visits | 98 (12.5%) | 86 (11.0%) | 556 (71.2%) | 22 (2.8%) | 19 (2.4%) | 781 |

**Figure 1.** Impacts of MyHealthNB on Costs: Descriptive Statistics of Likert Scores

## Part 4: Connection between MyHealthNB Impacts - Mediation Analysis

### A. Enablement to Empowerment to Outcomes Pathway

Table 1. Mediation Analysis Regression Results for Empowerment Pathways

| **Outcomes** | **Enablement → Outcome (β, p)** | **Empowerment → Outcome (β, p)** | **Enablement + Empowerment R²** | **Empowerment Mediation** |
| --- | --- | --- | --- | --- |
| **Engagement** | β=0.80, **p<0.001*** | β=0.19, **p<0.001*** | 0.61 | Yes |
| **Involvement** | β=0.92, **p<0.001*** | β=0.11, **p=0.001*** | 0.55 | Yes |
| **Cost** | β=0.57, **p<0.001*** | β=0.07, p=0.073 | 0.29 | No |

### A. Enablement to Stress to Outcomes Pathway

Table 1. Mediation Analysis Regression Results for Stress Pathways

| **Outcomes** | **Enablement → Outcome (β, p)** | **Stress → Outcome (β, p)** | **Enablement + Stress R²** | **Stress Mediation** |
| --- | --- | --- | --- | --- |
| **Engagement** | β=0.80, **p<0.001*** | β=–0.02, p=0.21 | 0.58 | No |
| **Involvement** | β=0.92, **p<0.001*** | β=0.00, p=0.96 | 0.53 | No |
| **Cost** | β=0.57, **p<0.001*** | β=–0.02, p=0.22 | 0.29 | No |

## Part 5: Predictors of MyHealthNB Impacts - Multiple Regression Analysis

### A. Multiple Regression Analysis with 16 Predictor Variables

**Table 1.** Multiple Regression Results for Predictors of MyHealthNB Impacts

| **Predictors** | **Racial Identity** | **Gender Identity** | **Digital Health Service Navigation Ease** | **Residence Length** | **Age** | **Ongoing Health Condition** | **Language Spoken** | **Location** | **MyHealthNB Access Format** | **Education Level** | **MyHealthNB Home Dashboard Use** | **MyHealthNB Use Frequency** | **Digital Literacy** | **Satisfaction with Provider Support of MyHealthNB** | **Having a Family Doctor** | **MyHealthNB Satisfaction Score** |
| --- | --- | --- | --- | --- | --- | --- | --- | --- | --- | --- | --- | --- | --- | --- | --- | --- |
|  | 0=White; 1=Non-White | 0=Male; 1=Female | 1=Hard; 4=Easy | 1=0-3 years; 4=20+years | 1=19-24; 8=85+ | 0=No; 1=Yes | 0=English only ; 1= French Speaking | 0=Major Centre 1=Rural Centre | 0=Web only; 1=App using | 0=No advanced degree; 1=Advanced degree | 0=No use; 1=Use | 1=Low; 3=High | 1=Not confident; 4=Very confident | 1=Dissatisfied; 5=Satisfied | 0=No; 1=Yes | 1=Dissatisfied; 5=Satisfied |
| **Outcomes of Interest** |  |  |  |  |  |  |  |  |  |  |  |  |  |  |  |  |
| **Ease of Accessing Health Information** | No | No | No | No | No | No | Yes (-0.114; p-value = 0.044) | No | No | Yes (0.202; p-value = 0.0009) | No | No | Yes (0.2081; p-value=<0.0000) | Yes (0.1077; p-value=<0.000) | Yes (0.2171; p-value = 0.0004) | Yes (0.3935; p-value=<0.0000) |
| **Awareness of Health Status** | No | No | No | No | No | No | No | No | No | No | Yes (0.1260; p-value=0.0199) | Yes (0.0858; p-value=0.0463) | Yes (0.2080; p-value=<0.0000) | Yes (0.0938; p-value = 0.0002) | Yes (0.3215; p-value = <0.0000) | Yes (0.3573; p-value=<0.0000) |
| **Understanding of Own Health** | No | No | No | No | No | No | No | No | Yes (0.1393; p-value = 0.0324) | No | Yes (0.1423; p-value=0.0320) | No | Yes (0.1810;p-value=0.0047) | Yes (0.1282; p-value = <0.0000) | Yes (0.3282; p-value= <0.000) | Yes (0.3629; p-value=<0.0000) |
| **Control Over Health Information** | No | No | No | No | No | No | No | No | No | No | No | Yes (0.1283; p-value=0.0265) | Yes (0.1575; p-value=0.0246) | Yes (0.1198; p-value= 0.0004) | Yes (0.4106; p-value = <0.0000) | Yes (0.2956; p-value=<0.0000) |
| **Patient Empowerment** | No | No | No | No | No | No | No | No | No | Yes (0.1509; p=0.0381) | Yes (0.2340; p=0.0002) | Yes (0.1381; p=0.0059) | Yes (0.1864; p=0.0023) | Yes (0.0924; p=0.0015) | Yes (0.2903; p-value = <0.0000) | Yes (0.4638; p=<0.0000) |
| **Patient Anxiety** | No | No | No | Yes (-0.1330; p=0.0294) | No | Yes (-0.374; p=0.0018) | No | No | No | Yes (-0.2700; p=0.0221) | No | No | Yes (-0.3660; p=0.0002) | No | Yes (-0.5400; p=<0.0000) | Yes (-0.4228; p=<0.0000) |
| **Engagement in Healthcare Behaviours** | No | No | No | No | No | No | No | No | Yes (0.1547; p-value = 0.0272) | No | Yes (0.2010; p-value=0.0048) | No | Yes (0.2655;p-value=0.0001) | Yes (0.15417; p-value= <0.0000) | Yes (0.4593; p-value = <0.0000) | Yes (0.3193; p-value=<0.0000) |
| **Engagement in Health Behaviours** | No | No | No | No | No | No | No | No | No | No | Yes (0.2503; p-value=0.0003) | No | Yes (0.3030; p-value=<0.0000) | Yes (0.1548; p-value=<0.0000) | Yes (0.3720; p-value = <0.0000) | Yes (0.2972; p-value=<0.0000) |
| **Preparedness for Appointments** | No | No | No | No | No | No | No | Yes (-0.281; p-value = 0.002) | No | No | No | Yes (0.2207; p-value=<0.0000) | Yes (0.2432; p-value=0.0002) | Yes (0.1377; p-value= <0.000) | Yes (0.4090); p-value = <0.0000) | Yes (0.2437; p-value=<0.0000) |
| **Communication with Providers** | No | No | No | No | No | No | No | Yes (-0.185; p-value = 0.049) | No | No | Yes (0.1441; p-value=0.0383) | Yes (0.2055; p-value=0.0002) | Yes (0.1615; p-value=0.0161) | Yes (0.1903; p-value = <0.0000) | Yes (0.4463; p-value= <0.0000) | Yes (0.2469; p-value=<0.0000) |
| **Engagement in Shared Decision Making** | No | No | No | No | No | No | No | No | No | Yes (0.1971; p-value = 0.0174) | Yes (0.1675; p-value=0.01976) | Yes (0.1736; p-value=0.0024) | Yes (0.2307; p-value=0.0009) | Yes (0.1708; p-valuie = <0.0000) | Yes (0.4530; p-value = <0.0000) | Yes (0.2717; p-value=<0.0000) |
| **Preparedness for a Health Emergency** | No | No | No | No | No | No | No | No | No | No | No | Yes (0.1670; p-value=0.0040) | Yes (0.1571; p-value=0.0253) | Yes (0.1606; p-value=<0.0000) | Yes (0.2638; p-value = 0.0021) | Yes (0.2307; p-value=<0.0000) |
| **Early Detection and Action** | No | No | No | No | No | No | No | No | No | Yes (0.1820; p-value = 0.0299) | No | Yes (0.2256; p-value=0.0001) | No | Yes (0.1568; p-value=<0.0000) | Yes (0.5281; p-value= <0.0000) | Yes (0.2974; p-value = <0.0000) |
| **Avoidance of Duplicate Tests** | No | No | No | No | Yes (-0.069; p-value =0.004) | Yes (-0.2042; p-value = 0.010) | Yes (0.1545; p-value= 0.035) | No | No | No | No | Yes (0.1885; p-value=0.0006) | No | Yes (0.1843; p-value=<0.0000) | Yes (0.2179; p-value = 0.0068) | Yes (0.1691; p-value=<0.0000) |
| **Appointment Efficiency** | No | No | No | No | No | No | No | No | No | No | No | Yes (0.1683; p-value=0.0033) | Yes (0.1710; p-value=0.0135) | Yes (0.2643; p-value=<0.0000) | Yes (0.4249; p-value = <0.0000) | Yes (0.2634; p-value=<0.0000) |
| **Healthcare Coordination** | No | No | Yes (0.0676; p-value = 0.0173) | No | No | No | No | No | No | No | Yes (0.1236; p-value=0.0389) | Yes (0.1236; p-value=0.0352) | No | Yes (0.2236; p-value=<0.0000) | Yes (0.3583; p-value = <0.0000) | Yes (0.2721; p-value=<0.0000) |
| **Overall Appointment Quality** | No | No | No | No | No | No | No | No | Yes (0.1385; p-value = 0.0377) | No | Yes (0.1845; p-value=0.0066) | Yes (0.1472; p-value=0.0064) | Yes (0.1536; p-value=0.0187) | Yes (0.2569; p-value=<0.0000) | Yes (0.4636; p-value = <0.0000) | Yes (0.2381; p-value=<0.0000) |
| **Decreased Personal Time Spent** | No | Yes (-0.1887; p=0.0341) | No | No | Yes (-0.0757; p=0.0067) | No | No | No | No | No | No | Yes (0.1861; p=0.0037) | No | Yes (0.0831; p=0.0253) | Yes (0.2774; p=0.0034) | Yes (0.2705; <0.0000) |
| **Decreased Calls to Providers** | No | No | No | No | No | No | No | No | No | No | No | No | Yes (0.2528; p=0.0014) | Yes (0.1108; p=0.0033) | Yes (0.3527; p=0.0003) | Yes (0.2672; p=<0.0000) |
| **Decreased Visits to Providers** | No | No | No | No | No | No | No | No | No | No | No | Yes (0.1564; p=0.0105) | No | Yes (0.0863; p=0.0146) | Yes (0.2445; p=0.0068) | Yes (0.2164; p=0.0002) |
| **Decreased ER Visits** | No | No | No | Yes (-0.098; p=0.0074) | No | No | No | Yes (0.1870; p=0.0252) | No | Yes (-0.1449; p=0.0405) | Yes (0.1223; p=0.0465) | Yes (0.1205; p=0.0145) | No | Yes (0.1084;p=0.0001) | Yes (0.1484; p=0.0393) | Yes (0.1393; p=0.0031) |

**Model For MyHealthNB’s Impact on Ease of Accessing Health Information**

**Model Summary**

R² = 0.308, Adjusted R² = 0.289

F(16, 601) = 16.68, p < .001

Standard Error = 0.61, n = 618

| **Predictor** | **B (Coeff.)** | **SE** | **t** | **p** | **95% CI** |
| --- | --- | --- | --- | --- | --- |
| Intercept | 1.569 | 0.269 | 5.84 | < .001 | [1.04, 2.10] |
| Age Range | -0.014 | 0.018 | -0.79 | .429 | [-0.05, 0.02] |
| Gender Identity | 0.063 | 0.058 | 1.08 | .280 | [-0.05, 0.18] |
| Racial Identity | -0.087 | 0.091 | -0.96 | .339 | [-0.26, 0.09] |
| Location Area | 0.006 | 0.072 | 0.08 | .933 | [-0.14, 0.15] |
| Residence Length | 0.035 | 0.031 | 1.11 | .269 | [-0.03, 0.10] |
| **Language** | **-0.114** | 0.057 | -2.02 | **.044** | [-0.23, -0.00] |
| **Education Level** | **0.202** | 0.061 | 3.32 | **< .001** | [0.08, 0.32] |
| **Family Doctor** | **0.217** | 0.062 | 3.51 | **< .001** | [0.10, 0.34] |
| Chronic Health | 0.031 | 0.061 | 0.50 | .618 | [-0.09, 0.15] |
| **Web Confidence** | **0.208** | 0.051 | 4.10 | **< .001** | [0.11, 0.31] |
| Navigation Ease | -0.009 | 0.020 | -0.44 | .661 | [-0.05, 0.03] |
| MyHealthNB Access Format | 0.007 | 0.052 | 0.13 | .893 | [-0.09, 0.11] |
| **Combined Satisfaction Score** | **0.393** | 0.040 | 9.77 | **< .001** | [0.31, 0.47] |
| Home Page Use | 0.066 | 0.053 | 1.25 | .211 | [-0.04, 0.17] |
| MyHealthNB Use Frequency | 0.029 | 0.042 | 0.70 | .484 | [-0.05, 0.11] |
| **Satisfaction with Provider Encouragement to Use MyHealthNB** | **0.108** | 0.024 | 4.45 | **< .001** | [0.06, 0.16] |

**Variance inflation factors (VIFs) for Predictors in the Model Examining MyHealthNB’s Impact on Ease of Accessing Health Information**

| **Predictor** | **VIF** |
| --- | --- |
| Age Range | 1.45 |
| Gender Identity | 1.07 |
| Racial Identity | 1.19 |
| Location Area | 1.12 |
| Residence Length | 1.22 |
| Language | 1.14 |
| Education Level | 1.11 |
| Family Doctor | 1.11 |
| Chronic Health | 1.13 |
| Web Confidence | 1.12 |
| Navigation Ease | 1.18 |
| MyHealthNB Access Format | 1.12 |
| Combined Satisfaction Score | 1.21 |
| Home Page Use | 1.16 |
| MyHealthNB Use Frequency | 1.28 |
| Satisfaction with Provider Encouragement to Use MyHealthNB | 1.27 |

**Model for MyHealthNB’s Impact on Awareness of Health Status**

**Model Summary:**

R² = 0.297, Adjusted R² = 0.278

F(16, 602) = 15.86, p < .001

Standard Error = 0.62, n = 619

| **Predictor** | **B (Coeff.)** | **SE** | **t** | **p** | **95% CI** |
| --- | --- | --- | --- | --- | --- |
| Intercept | 1.393 | 0.276 | 5.06 | < .001 | [0.85, 1.93] |
| Age Range | 0.011 | 0.019 | 0.60 | .546 | [–0.03, 0.05] |
| Gender Identity | 0.062 | 0.060 | 1.03 | .302 | [–0.06, 0.18] |
| Racial Identity | –0.113 | 0.093 | –1.22 | .223 | [–0.30, 0.07] |
| Location Area | 0.053 | 0.074 | 0.73 | .469 | [–0.09, 0.20] |
| Residence Length | 0.045 | 0.032 | 1.41 | .159 | [–0.02, 0.11] |
| Language | –0.030 | 0.058 | –0.52 | .600 | [–0.14, 0.08] |
| Education Level | 0.109 | 0.062 | 1.75 | .081 | [–0.01, 0.23] |
| **Family Doctor** | **0.322** | 0.063 | **5.08** | **< .001** | **[0.20, 0.45]** |
| Chronic Health | –0.010 | 0.063 | –0.15 | .880 | [–0.13, 0.11] |
| **Web Confidence** | **0.208** | 0.052 | **4.00** | **< .001** | **[0.11, 0.31]** |
| Navigation Ease | 0.004 | 0.021 | 0.18 | .859 | [–0.04, 0.04] |
| MyHealthNB Access Format | 0.006 | 0.053 | 0.11 | .912 | [–0.10, 0.11] |
| **Combined Satisfaction Score** | **0.357** | 0.041 | **8.65** | **< .001** | **[0.28, 0.44]** |
| **Home Page Use** | **0.126** | **0.054** | **2.34** | **.020** | **[0.02, 0.23]** |
| **MyHealthNB Use Frequency** | **0.086** | **0.043** | **2.00** | **.046** | **[0.00, 0.17]** |
| **Satisfaction with Provider Encouragement to Use MyHealthNB** | **0.094** | 0.025 | **3.78** | **< .001** | **[0.05, 0.14]** |

**Model for MyHealthNB’s Impact on Understanding of Own Health**

**Model Summary**

R² = 0.255, Adjusted R² = 0.235

F(16, 600) = 12.82, p < .001

Standard Error = 0.76, n = 617

| **Predictor** | **B (Coeff.)** | **SE** | **t** | **p** | **95% CI** |
| --- | --- | --- | --- | --- | --- |
| Intercept | 0.930 | 0.338 | 2.75 | .006 | [0.27, 1.60] |
| Age Range | –0.007 | 0.023 | –0.30 | .762 | [–0.05, 0.04] |
| Gender Identity | –0.027 | 0.073 | –0.37 | .710 | [–0.17, 0.12] |
| Racial Identity | –0.078 | 0.114 | –0.69 | .492 | [–0.30, 0.15] |
| Location Area | 0.075 | 0.090 | 0.83 | .408 | [–0.10, 0.25] |
| Residence Length | 0.048 | 0.040 | 1.22 | .224 | [–0.03, 0.13] |
| Language | –0.094 | 0.071 | –1.33 | .185 | [–0.23, 0.05] |
| Education Level | 0.101 | 0.076 | 1.32 | .188 | [–0.05, 0.25] |
| **Family Doctor** | **0.328** | 0.078 | **4.23** | **< .001** | **[0.18, 0.48]** |
| Chronic Health | 0.036 | 0.077 | 0.46 | .644 | [–0.12, 0.19] |
| **Web Confidence** | **0.181** | 0.064 | **2.84** | **.005** | **[0.06, 0.31]** |
| Navigation Ease | 0.035 | 0.025 | 1.40 | .161 | [–0.01, 0.09] |
| *MyHealthNB Access Format* | *0.139* | 0.065 | *2.14* | *.032* | *[0.01, 0.27]* |
| **Combined Satisfaction Score** | **0.363** | 0.051 | **7.11** | **< .001** | **[0.26, 0.46]** |
| **Home Page Use** | **0.142** | **0.066** | **2.15** | **.032** | **[0.01, 0.27]** |
| MyHealthNB Use Frequency | 0.101 | 0.053 | 1.92 | .055 | [–0.00, 0.20] |
| **Satisfaction with Provider Encouragement to Use MyHealthNB** | **0.128** | 0.030 | **4.21** | **< .001** | **[0.07, 0.19]** |

**Model for MyHealthNB’s Impact on Control**

**Model Summary**
R² = 0.212, Adjusted R² = 0.191
F (16, 603) = 10.15, p < .001
Standard Error = 0.84, n = 620

| **Predictor** | **B (Coeff.)** | **SE** | **t** | **p** | **95 % CI** |
| --- | --- | --- | --- | --- | --- |
| **Intercept** | **1.416** | 0.370 | **3.82** | **< .001** | **[0.69, 2.14]** |
| Age Range | –0.044 | 0.025 | –1.77 | .077 | [–0.09, 0.00] |
| Gender Identity | 0.067 | 0.080 | 0.83 | .405 | [–0.09, 0.22] |
| Racial Identity | 0.012 | 0.125 | 0.10 | .923 | [–0.23, 0.26] |
| Location Area | –0.159 | 0.099 | –1.61 | .107 | [–0.35, 0.03] |
| Residence Length | –0.009 | 0.043 | –0.22 | .827 | [–0.09, 0.08] |
| Language | 0.086 | 0.078 | 1.11 | .268 | [–0.07, 0.24] |
| Education Level | 0.121 | 0.084 | 1.45 | .148 | [–0.04, 0.29] |
| **Family Doctor** | **0.411** | 0.085 | **4.82** | **< .001** | **[0.24, 0.58]** |
| Chronic Health | –0.074 | 0.084 | –0.88 | .381 | [–0.24, 0.09] |
| *Web Confidence* | *0.157* | 0.070 | *2.25* | *.025* | *[0.02, 0.29]* |
| Navigation Ease | 0.034 | 0.028 | 1.24 | .215 | [–0.02, 0.09] |
| MyHealthNB Access Format | 0.023 | 0.071 | 0.32 | .749 | [–0.12, 0.16] |
| **Combined Satisfaction Score** | **0.296** | 0.056 | **5.33** | **< .001** | **[0.19, 0.40]** |
| Home Page Use | 0.129 | 0.072 | 1.77 | .076 | [–0.01, 0.27] |
| *MyHealthNB Use Frequency* | *0.128* | 0.058 | *2.22* | *.026* | *[0.02, 0.24]* |
| **Satisfaction with Provider Encouragement to Use MyHealthNB** | **0.120** | 0.033 | **3.59** | **< .001** | **[0.05, 0.19]** |

**Model for MyHealthNB’s Impact on Engagement in Healthcare Behaviors**

**Model Summary**
R² = 0.263, Adjusted R² = 0.244
F(16, 601) = 13.43, p < .001
Standard Error = 0.82, n = 618

| **Predictor** | **B (Coeff.)** | **SE** | **t** | **p** | **95 % CI** |
| --- | --- | --- | --- | --- | --- |
| Intercept | 0.716 | 0.363 | 1.97 | .049 | [0.00, 1.43] |
| Age Range | –0.007 | 0.025 | –0.28 | .783 | [–0.06, 0.04] |
| Gender Identity | 0.013 | 0.079 | 0.16 | .873 | [–0.14, 0.17] |
| Racial Identity | 0.020 | 0.122 | 0.17 | .868 | [–0.22, 0.26] |
| Location Area | –0.082 | 0.097 | –0.84 | .399 | [–0.27, 0.11] |
| Residence Length | –0.009 | 0.042 | –0.21 | .833 | [–0.09, 0.07] |
| Language | –0.026 | 0.076 | –0.34 | .737 | [–0.18, 0.12] |
| Education Level | 0.092 | 0.082 | 1.12 | .263 | [–0.07, 0.25] |
| **Family Doctor** | **0.459** | 0.083 | **5.52** | **< .001** | **[0.30, 0.62]** |
| Chronic Health | –0.036 | 0.083 | –0.44 | .663 | [–0.20, 0.13] |
| **Web Confidence** | **0.266** | 0.068 | **3.88** | **< .001** | **[0.13, 0.40]** |
| Navigation Ease | 0.046 | 0.027 | 1.70 | .090 | [–0.01, 0.10] |
| *MyHealthNB Access Format* | *0.155* | 0.070 | *2.21* | *.027* | *[0.02, 0.29]* |
| **Combined Satisfaction Score** | **0.319** | 0.054 | **5.87** | **< .001** | **[0.21, 0.43]** |
| **Home Page Use** | **0.201** | 0.071 | **2.83** | **.005** | **[0.06, 0.34]** |
| MyHealthNB Use Frequency | 0.052 | 0.057 | 0.91 | .362 | [–0.06, 0.16] |
| **Satisfaction with Provider Encouragement to Use MyHealthNB** | **0.154** | 0.033 | **4.71** | **< .001** | **[0.09, 0.22]** |

**Model for MyHealthNB’s Impact on Engagement in Health Behaviors**

**Model Summary**
R² = 0.255, Adjusted R² = 0.235
F (16, 599) = 12.82, p < .001
Standard Error = 0.80, n = 616

| **Predictor** | **B (Coeff.)** | **SE** | **t** | **p** | **95 % CI** |
| --- | --- | --- | --- | --- | --- |
| *Intercept* | 0.696 | 0.353 | 1.97 | *.049* | [0.00, 1.39] |
| Age Range | –0.018 | 0.024 | –0.77 | .443 | [–0.07, 0.03] |
| Gender Identity | –0.077 | 0.077 | –1.00 | .317 | [–0.23, 0.07] |
| Racial Identity | 0.035 | 0.119 | 0.30 | .767 | [–0.20, 0.27] |
| Location Area | –0.067 | 0.095 | –0.70 | .481 | [–0.25, 0.12] |
| Residence Length | 0.010 | 0.041 | 0.25 | .803 | [–0.07, 0.09] |
| Language | 0.058 | 0.074 | 0.79 | .432 | [–0.09, 0.20] |
| Education Level | 0.033 | 0.080 | 0.41 | .682 | [–0.12, 0.19] |
| **Family Doctor** | **0.372** | 0.081 | **4.60** | **< .001** | **[0.21, 0.53]** |
| Chronic Health | 0.112 | 0.081 | 1.38 | .169 | [–0.05, 0.27] |
| **Web Confidence** | **0.303** | 0.067 | **4.55** | **< .001** | **[0.17, 0.43]** |
| Navigation Ease | 0.041 | 0.027 | 1.55 | .123 | [–0.01, 0.09] |
| MyHealthNB Access Format | 0.012 | 0.068 | 0.18 | .860 | [–0.12, 0.15] |
| **Combined Satisfaction Score** | **0.297** | 0.053 | **5.61** | **< .001** | **[0.19, 0.40]** |
| **Home Page Use** | **0.250** | 0.069 | **3.61** | **.001** | **[0.11, 0.39]** |
| MyHealthNB Use Frequency | 0.040 | 0.055 | 0.73 | .466 | [–0.07, 0.15] |
| **Satisfaction with Provider Encouragement to Use MyHealthNB** | **0.155** | 0.032 | **4.86** | **< .001** | **[0.09, 0.22]** |

**Model for MyHealthNB’s Impact on Appointment Preparedness**

**Model Summary**
R² = 0.249, Adjusted R² = 0.229
F (16, 603) = 12.48, p < .001
Standard Error = 0.78, n = 620

| **Predictor** | **B (Coeff.)** | **SE** | **t** | **p** | **95 % CI** |
| --- | --- | --- | --- | --- | --- |
| *Intercept* | 0.789 | 0.346 | 2.28 | *.023* | [0.11, 1.47] |
| Age Range | 0.027 | 0.023 | 1.15 | .252 | [–0.02, 0.07] |
| Gender Identity | –0.014 | 0.075 | –0.19 | .851 | [–0.16, 0.13] |
| Racial Identity | –0.115 | 0.117 | –0.98 | .326 | [–0.34, 0.11] |
| **Location Area** | **–0.281** | 0.092 | **–3.06** | **.002** | **[–0.46, –0.10]** |
| Residence Length | 0.044 | 0.040 | 1.09 | .274 | [–0.04, 0.12] |
| Language | –0.027 | 0.073 | –0.38 | .707 | [–0.17, 0.12] |
| Education Level | 0.123 | 0.078 | 1.58 | .115 | [–0.03, 0.28] |
| **Family Doctor** | **0.409** | 0.080 | **5.13** | **< .001** | **[0.25, 0.57]** |
| Chronic Health | 0.118 | 0.079 | 1.49 | .136 | [–0.04, 0.27] |
| **Web Confidence** | **0.243** | 0.065 | **3.73** | **< .001** | **[0.12, 0.37]** |
| Navigation Ease | 0.032 | 0.026 | 1.25 | .213 | [–0.02, 0.08] |
| MyHealthNB Access Format | 0.030 | 0.066 | 0.45 | .653 | [–0.10, 0.16] |
| **Combined Satisfaction Score** | **0.244** | 0.052 | **4.70** | **< .001** | **[0.14, 0.35]** |
| Home Page Use | 0.127 | 0.068 | 1.88 | .060 | [–0.01, 0.26] |
| **MyHealthNB Use Frequency** | **0.221** | 0.054 | **4.10** | **< .001** | **[0.11, 0.33]** |
| **Satisfaction with Provider Encouragement to Use MyHealthNB** | **0.138** | 0.031 | **4.42** | **< .001** | **[0.08, 0.20]** |

**Model for MyHealthNB’s Impact on Communication with Providers**

**Model Summary**
R² = 0.264, Adjusted R² = 0.245
F (16, 603) = 13.52, p < .001
Standard Error = 0.80, n = 620

| **Predictor** | **B (Coeff.)** | **SE** | **t** | **p** | **95 % CI** |
| --- | --- | --- | --- | --- | --- |
| *Intercept* | 0.888 | 0.355 | 2.50 | *.013* | [0.19, 1.58] |
| Age Range | 0.031 | 0.024 | 1.27 | .204 | [–0.02, 0.08] |
| Gender Identity | –0.047 | 0.077 | –0.61 | .541 | [–0.20, 0.10] |
| Racial Identity | –0.010 | 0.120 | –0.08 | .934 | [–0.25, 0.23] |
| *Location Area* | *–0.185* | 0.094 | *–1.96* | *.050* | [–0.37, 0.00] |
| Residence Length | 0.018 | 0.042 | 0.43 | .668 | [–0.06, 0.10] |
| Language | 0.067 | 0.074 | 0.90 | .369 | [–0.08, 0.21] |
| Education Level | 0.102 | 0.080 | 1.27 | .205 | [–0.06, 0.26] |
| **Family Doctor** | **0.446** | 0.082 | **5.46** | **< .001** | **[0.29, 0.61]** |
| Chronic Health | 0.073 | 0.081 | 0.91 | .365 | [–0.09, 0.23] |
| *Web Confidence* | *0.162* | 0.067 | *2.41* | *.016* | [0.03, 0.29] |
| Navigation Ease | 0.046 | 0.026 | 1.75 | .081 | [–0.01, 0.10] |
| MyHealthNB Access Format | 0.089 | 0.068 | 1.30 | .193 | [–0.04, 0.22] |
| **Combined Satisfaction Score** | **0.247** | 0.053 | **4.64** | **< .001** | **[0.14, 0.35]** |
| *Home Page Use* | *0.144* | 0.069 | *2.08* | *.038* | [0.01, 0.28] |
| **MyHealthNB Use Frequency** | **0.206** | 0.055 | **3.72** | **< .001** | **[0.10, 0.31]** |
| **Satisfaction with Provider Encouragement to Use MyHealthNB** | **0.190** | 0.032 | **5.95** | **< .001** | **[0.13, 0.25]** |

**Model for MyHealthNB’s Impact on Shared Decision Making**

**Model Summary**
R² = 0.252, Adjusted R² = 0.233
F (16, 601) = 12.68, p < .001
Standard Error = 0.82, n = 618

| **Predictor** | **B (Coeff.)** | **SE** | **t** | **p** | **95 % CI** |
| --- | --- | --- | --- | --- | --- |
| *Intercept* | 0.880 | 0.366 | 2.40 | *.017* | [0.16, 1.60] |
| Age Range | 0.002 | 0.025 | 0.08 | .939 | [–0.05, 0.05] |
| Gender Identity | –0.110 | 0.079 | –1.39 | .166 | [–0.27, 0.05] |
| Racial Identity | –0.193 | 0.123 | –1.56 | .118 | [–0.44, 0.05] |
| Location Area | –0.174 | 0.097 | –1.79 | .074 | [–0.37, 0.02] |
| Residence Length | –0.000 | 0.043 | –0.01 | .995 | [–0.08, 0.08] |
| Language | 0.030 | 0.077 | 0.40 | .692 | [–0.12, 0.18] |
| *Education Level* | *0.197* | 0.083 | *2.39* | *.017* | [0.03, 0.36] |
| **Family Doctor** | **0.453** | 0.085 | **5.36** | **< .001** | **[0.29, 0.62]** |
| Chronic Health | 0.109 | 0.083 | 1.31 | .190 | [–0.05, 0.27] |
| **Web Confidence** | **0.231** | 0.069 | **3.34** | **< .001** | **[0.10, 0.37]** |
| Navigation Ease | –0.004 | 0.027 | –0.14 | .888 | [–0.06, 0.05] |
| MyHealthNB Access Format | 0.092 | 0.070 | 1.31 | .192 | [–0.05, 0.23] |
| **Combined Satisfaction Score** | **0.272** | 0.055 | **4.95** | **< .001** | **[0.16, 0.38]** |
| *Home Page Use* | *0.168* | 0.072 | *2.34* | *.020* | [0.03, 0.31] |
| **MyHealthNB Use Frequency** | **0.174** | 0.057 | **3.04** | **.002** | **[0.06, 0.29]** |
| **Satisfaction with Provider Encouragement to Use MyHealthNB** | **0.171** | 0.033 | **5.18** | **< .001** | **[0.11, 0.24]** |

**Model for MyHealthNB’s Impact on Emergency Preparedness**

**Model Summary**

R² = 0.206, Adjusted R² = 0.185

F (16, 602) = 9.76, p < .001

Standard Error = 0.84, n = 619

| **Predictor** | **B (Coeff.)** | **SE** | **t** | **p** | **95 % CI** |
| --- | --- | --- | --- | --- | --- |
| *Intercept* | 1.272 | 0.371 | 3.43 | *.001* | [0.54, 2.00] |
| Age Range | –0.030 | 0.025 | –1.21 | .227 | [–0.08, 0.02] |
| Gender Identity | –0.105 | 0.080 | –1.30 | .193 | [–0.26, 0.05] |
| Racial Identity | –0.004 | 0.125 | –0.03 | .977 | [–0.25, 0.24] |
| Location Area | –0.088 | 0.099 | –0.88 | .377 | [–0.28, 0.11] |
| Residence Length | –0.001 | 0.043 | –0.01 | .989 | [–0.09, 0.08] |
| Language | 0.101 | 0.078 | 1.30 | .195 | [–0.05, 0.25] |
| Education Level | 0.063 | 0.084 | 0.75 | .453 | [–0.10, 0.23] |
| **Family Doctor** | **0.264** | 0.085 | **3.09** | **.002** | **[0.10, 0.43]** |
| Chronic Health | 0.078 | 0.085 | 0.92 | .356 | [–0.09, 0.24] |
| *Web Confidence* | *0.157* | 0.070 | *2.24* | *.025* | [0.02, 0.29] |
| Navigation Ease | 0.052 | 0.028 | 1.86 | .063 | [–0.00, 0.11] |
| MyHealthNB Access Format | 0.066 | 0.071 | 0.93 | .355 | [–0.07, 0.21] |
| **Combined Satisfaction Score** | **0.231** | 0.056 | **4.14** | **< .001** | **[0.12, 0.34]** |
| Home Page Use | 0.142 | 0.073 | 1.95 | .051 | [–0.00, 0.28] |
| **MyHealthNB Use Frequency** | **0.167** | 0.058 | **2.89** | **.004** | **[0.05, 0.28]** |
| **Satisfaction with Provider Encouragement to Use MyHealthNB** | **0.161** | 0.033 | **4.80** | **< .001** | **[0.09, 0.23]** |

**Model for MyHealthNB’s Impact on Early Detection and Action**

**Model Summary**
R² = 0.252 Adjusted R² = 0.232
F (16, 601) = 12.66, *p* < .001
Standard Error = 0.83, *n* = 618

| **Predictor** | **B (Coeff.)** | **SE** | **t** | **p** | **95 % CI** |
| --- | --- | --- | --- | --- | --- |
| **Intercept** | **1.186** | 0.370 | **3.20** | **.001** | **[0.46, 1.91]** |
| Age Range | 0.016 | 0.025 | 0.64 | .522 | [-0.03, 0.07] |
| Gender Identity | –0.119 | 0.080 | –1.49 | .138 | [-0.28, 0.04] |
| Racial Identity | –0.045 | 0.125 | –0.36 | .721 | [-0.29, 0.20] |
| Location Area | –0.062 | 0.099 | –0.62 | .534 | [-0.26, 0.13] |
| Residence Length | –0.036 | 0.043 | –0.83 | .406 | [-0.12, 0.05] |
| Language | 0.110 | 0.078 | 1.42 | .156 | [-0.04, 0.26] |
| *Education Level* | *0.182* | 0.084 | *2.18* | *.030* | *[0.02, 0.35]* |
| **Family Doctor** | **0.528** | 0.085 | **6.19** | **< .001** | **[0.36, 0.70]** |
| Chronic Health | –0.090 | 0.084 | –1.07 | .285 | [-0.26, 0.08] |
| Web Confidence | 0.094 | 0.070 | 1.35 | .179 | [-0.04, 0.23] |
| Navigation Ease | 0.039 | 0.028 | 1.40 | .163 | [-0.02, 0.09] |
| MyHealthNB Access Format | 0.101 | 0.071 | 1.41 | .158 | [-0.04, 0.24] |
| **Combined Satisfaction Score** | **0.297** | 0.056 | **5.36** | **< .001** | **[0.19, 0.41]** |
| Home Page Use | 0.045 | 0.073 | 0.63 | .531 | [-0.10, 0.19] |
| **MyHealthNB Use Frequency** | **0.226** | 0.058 | **3.91** | **< .001** | **[0.11, 0.34]** |
| **Satisfaction with Provider Encouragement to Use MyHealthNB** | **0.157** | 0.033 | **4.70** | **< .001** | **[0.09, 0.22]** |

**Model for MyHealthNB’s Impact on Duplicate Avoidance**

**Model Summary**
R² = 0.227 Adjusted R² = 0.207
F (16, 602) = 11.07, *p* < .001
Standard Error = 0.79, *n* = 619

| **Predictor** | **B (Coeff.)** | **SE** | **t** | **p** | **95 % CI** |
| --- | --- | --- | --- | --- | --- |
| **Intercept** | **2.388** | 0.348 | **6.86** | **< .001** | **[1.70, 3.07]** |
| **Age Range** | **–0.069** | 0.024 | **–2.92** | **.004** | **[–0.12, –0.02]** |
| Gender Identity | 0.029 | 0.075 | 0.39 | .698 | [–0.12, 0.18] |
| Racial Identity | 0.036 | 0.118 | 0.30 | .762 | [–0.20, 0.27] |
| Location Area | 0.023 | 0.093 | 0.24 | .807 | [–0.16, 0.21] |
| Residence Length | –0.040 | 0.041 | –0.99 | .325 | [–0.12, 0.04] |
| *Language* | *0.155* | 0.073 | *2.11* | *.035* | *[0.01, 0.30]* |
| Education Level | 0.064 | 0.079 | 0.81 | .417 | [–0.09, 0.22] |
| **Family Doctor** | **0.218** | 0.080 | **2.72** | **.007** | **[0.06, 0.38]** |
| *Chronic Health* | *–0.204* | 0.079 | *–2.57* | *.010* | *[–0.36, –0.05]* |
| Web Confidence | –0.031 | 0.066 | –0.47 | .638 | [–0.16, 0.10] |
| Navigation Ease | 0.019 | 0.026 | 0.75 | .456 | [–0.03, 0.07] |
| MyHealthNB Access Format | 0.063 | 0.067 | 0.94 | .350 | [–0.07, 0.19] |
| **Combined Satisfaction Score** | **0.169** | 0.052 | **3.24** | **.001** | **[0.07, 0.27]** |
| Home Page Use | 0.077 | 0.068 | 1.13 | .260 | [–0.06, 0.21] |
| **MyHealthNB Use Frequency** | **0.188** | 0.054 | **3.47** | **< .001** | **[0.08, 0.30]** |
| **Satisfaction with Provider Encouragement to Use MyHealthNB** | **0.184** | 0.031 | **5.87** | **< .001** | **[0.12, 0.25]** |

**Model for MyHealthNB’s Impact on Appointment Efficiency**

**Model Summary**
R² = 0.286 Adjusted R² = 0.267
F (16, 602) = 15.08, *p* < .001
Standard Error = 0.82, *n* = 619

| **Predictor** | **B (Coeff.)** | **SE** | **t** | **p** | **95 % CI** |
| --- | --- | --- | --- | --- | --- |
| Intercept | 0.550 | 0.366 | 1.50 | .133 | [-0.17, 1.27] |
| Age Range | 0.012 | 0.025 | 0.49 | .627 | [-0.04, 0.06] |
| Gender Identity | –0.006 | 0.079 | –0.08 | .938 | [-0.16, 0.15] |
| Racial Identity | –0.038 | 0.123 | –0.31 | .757 | [-0.28, 0.20] |
| Location Area | –0.093 | 0.098 | –0.95 | .341 | [-0.28, 0.10] |
| Residence Length | 0.005 | 0.043 | 0.11 | .912 | [-0.08, 0.09] |
| Language | 0.049 | 0.077 | 0.64 | .524 | [-0.10, 0.20] |
| Education Level | 0.106 | 0.083 | 1.28 | .202 | [-0.06, 0.27] |
| **Family Doctor** | **0.425** | 0.084 | **5.05** | **< .001** | **[0.26, 0.59]** |
| Chronic Health | 0.002 | 0.083 | 0.02 | .982 | [-0.16, 0.17] |
| *Web Confidence* | *0.171* | 0.069 | *2.48* | *.013* | *[0.04, 0.31]* |
| Navigation Ease | 0.027 | 0.027 | 0.99 | .322 | [-0.03, 0.08] |
| MyHealthNB Access Format | 0.065 | 0.070 | 0.93 | .352 | [-0.07, 0.20] |
| **Combined Satisfaction Score** | **0.263** | 0.055 | **4.81** | **< .001** | **[0.16, 0.37]** |
| Home Page Use | 0.126 | 0.072 | 1.77 | .078 | [-0.01, 0.27] |
| **MyHealthNB Use Frequency** | **0.168** | 0.057 | **2.95** | **.003** | **[0.06, 0.28]** |
| **Satisfaction with Provider Encouragement to Use MyHealthNB** | **0.264** | 0.033 | **8.02** | **< .001** | **[0.20, 0.33]** |

**Model for MyHealthNB’s Impact on Healthcare Coordination**

**Model Summary**
R² = 0.259 Adjusted R² = 0.239
F (16, 597) = 13.03, *p* < .001
Standard Error = 0.85, *n* = 614

| **Predictor** | **B (Coeff.)** | **SE** | **t** | **p** | **95 % CI** |
| --- | --- | --- | --- | --- | --- |
| *Intercept* | 0.907 | 0.377 | 2.41 | *.016* | [0.17, 1.65] |
| Age Range | –0.018 | 0.026 | –0.72 | .474 | [–0.07, 0.03] |
| Gender Identity | 0.004 | 0.082 | 0.05 | .963 | [–0.16, 0.16] |
| Racial Identity | –0.020 | 0.127 | –0.16 | .875 | [–0.27, 0.23] |
| Location Area | –0.123 | 0.101 | –1.21 | .225 | [–0.32, 0.08] |
| Residence Length | 0.009 | 0.044 | 0.21 | .830 | [–0.08, 0.10] |
| Language | 0.150 | 0.079 | 1.90 | .058 | [–0.01, 0.30] |
| Education Level | 0.043 | 0.085 | 0.51 | .610 | [–0.12, 0.21] |
| **Family Doctor** | **0.358** | 0.086 | **4.14** | **< .001** | **[0.19, 0.53]** |
| Chronic Health | –0.022 | 0.086 | –0.25 | .800 | [–0.19, 0.15] |
| Web Confidence | 0.129 | 0.072 | 1.80 | .072 | [–0.01, 0.27] |
| *Navigation Ease* | *0.068* | 0.028 | *2.39* | *.017* | *[0.01, 0.12]* |
| MyHealthNB Access Format | 0.105 | 0.073 | 1.45 | .147 | [–0.04, 0.25] |
| **Combined Satisfaction Score** | **0.272** | 0.056 | **4.82** | **< .001** | **[0.16, 0.38]** |
| *Home Page Use* | *0.153* | 0.074 | *2.07* | *.039* | *[0.01, 0.30]* |
| *MyHealthNB Use Frequency* | *0.124* | 0.059 | *2.11* | *.035* | *[0.01, 0.24]* |
| **Satisfaction with Provider Encouragement** | **0.224** | 0.034 | **6.60** | **< .001** | **[0.16, 0.29]** |

**Model for MyHealthNB’s Impact on Overall Appointment Quality**

**Model Summary**
R² = 0.315, Adjusted R² = 0.296
F (16, 601) = 17.24, *p* < .001
Standard Error = 0.78, *n* = 618

| **Predictor** | **B (Coeff.)** | **SE** | **t** | **p** | **95 % CI** |
| --- | --- | --- | --- | --- | --- |
| Intercept | 0.652 | 0.346 | 1.89 | .059 | [-0.03, 1.33] |
| Age Range | 0.023 | 0.024 | 1.00 | .320 | [-0.02, 0.07] |
| Gender Identity | –0.011 | 0.075 | –0.14 | .886 | [-0.16, 0.14] |
| Racial Identity | 0.117 | 0.116 | 1.00 | .317 | [-0.11, 0.35] |
| Location Area | –0.064 | 0.092 | –0.69 | .488 | [-0.24, 0.12] |
| Residence Length | 0.004 | 0.041 | 0.09 | .925 | [-0.08, 0.08] |
| Language | 0.099 | 0.072 | 1.37 | .172 | [-0.04, 0.24] |
| Education Level | 0.106 | 0.078 | 1.35 | .176 | [-0.05, 0.26] |
| **Family Doctor** | **0.464** | 0.079 | **5.83** | **< .001** | **[0.31, 0.62]** |
| Chronic Health | –0.093 | 0.079 | –1.17 | .242 | [-0.25, 0.06] |
| *Web Confidence* | *0.154* | 0.065 | *2.36* | *.019* | *[0.03, 0.28]* |
| Navigation Ease | 0.032 | 0.026 | 1.25 | .213 | [-0.02, 0.08] |
| *MyHealthNB Access Format* | *0.138* | 0.066 | *2.08* | *.038* | *[0.01, 0.27]* |
| **Combined Satisfaction Score** | **0.238** | 0.052 | **4.60** | **< .001** | **[0.14, 0.34]** |
| **Home Page Use** | **0.184** | 0.068 | **2.73** | **.007** | **[0.05, 0.32]** |
| **MyHealthNB Use Frequency** | **0.147** | 0.054 | **2.74** | **.006** | **[0.04, 0.25]** |
| **Satisfaction with Provider Encouragement to Use MyHealthNB** | **0.257** | 0.031 | **8.26** | **< .001** | **[0.20, 0.32]** |

**Model for MyHealthNB’s Impact on Personal Time**

**Model Summary**
R² = 0.139 Adjusted R² = 0.116
F (16, 600) = 6.07, *p* < .001
Standard Error = 0.92, *n* = 617

| **Predictor** | **B (Coeff.)** | **SE** | **t** | **p** | **95 % CI** |
| --- | --- | --- | --- | --- | --- |
| **Intercept** | **1.848** | 0.410 | **4.51** | **< .001** | **[1.04, 2.65]** |
| **Age Range** | **–0.076** | 0.028 | **–2.72** | **.007** | **[–0.13, –0.02]** |
| *Gender Identity* | *–0.189* | 0.089 | *–2.12* | *.034* | *[–0.36, –0.01]* |
| Racial Identity | –0.061 | 0.138 | –0.44 | .661 | [–0.33, 0.21] |
| Location Area | 0.028 | 0.109 | 0.26 | .799 | [–0.19, 0.24] |
| Residence Length | 0.036 | 0.048 | 0.75 | .456 | [–0.06, 0.13] |
| Language | –0.013 | 0.086 | –0.15 | .883 | [–0.18, 0.16] |
| Education Level | 0.085 | 0.092 | 0.92 | .360 | [–0.10, 0.27] |
| **Family Doctor** | **0.277** | 0.094 | **2.94** | **.003** | **[0.09, 0.46]** |
| Chronic Health | –0.040 | 0.093 | –0.43 | .665 | [–0.22, 0.14] |
| Web Confidence | 0.145 | 0.077 | 1.87 | .062 | [–0.01, 0.30] |
| Navigation Ease | –0.032 | 0.031 | –1.05 | .296 | [–0.09, 0.03] |
| MyHealthNB Access Format | 0.064 | 0.079 | 0.82 | .415 | [–0.09, 0.22] |
| **Combined Satisfaction Score** | **0.271** | 0.061 | **4.40** | **< .001** | **[0.15, 0.39]** |
| Home Page Use | 0.081 | 0.080 | 1.01 | .312 | [–0.08, 0.24] |
| **MyHealthNB Use Frequency** | **0.186** | 0.064 | **2.92** | **.004** | **[0.06, 0.31]** |
| *Satisfaction with Provider Encouragement* | *0.083* | 0.037 | *2.24* | *.025* | *[0.01, 0.16]* |

**Model for MyHealthNB’s Impact on Calls to Providers**

**Model Summary**
R² = 0.137 Adjusted R² = 0.114
F (16, 602) = 5.99, *p* < .001
Standard Error = 0.94, *n* = 619

| **Predictor** | **B (Coeff.)** | **SE** | **t** | **p** | **95 % CI** |
| --- | --- | --- | --- | --- | --- |
| **Intercept** | **1.276** | 0.416 | **3.06** | **.002** | **[0.46, 2.09]** |
| Age Range | –0.039 | 0.028 | –1.37 | .171 | [–0.09, 0.02] |
| Gender Identity | –0.021 | 0.090 | –0.24 | .814 | [–0.20, 0.16] |
| Racial Identity | 0.034 | 0.140 | 0.24 | .807 | [–0.24, 0.31] |
| Location Area | 0.061 | 0.111 | 0.55 | .583 | [–0.16, 0.28] |
| Residence Length | 0.037 | 0.049 | 0.76 | .448 | [–0.06, 0.13] |
| Language | –0.007 | 0.087 | –0.08 | .936 | [–0.18, 0.16] |
| Education Level | 0.000 | 0.094 | 0.00 | .996 | [–0.18, 0.19] |
| **Family Doctor** | **0.353** | 0.096 | **3.68** | **< .001** | **[0.16, 0.54]** |
| Chronic Health | –0.113 | 0.095 | –1.19 | .236 | [–0.30, 0.07] |
| **Web Confidence** | **0.253** | 0.079 | **3.22** | **.001** | **[0.10, 0.41]** |
| Navigation Ease | –0.008 | 0.031 | –0.26 | .792 | [–0.07, 0.05] |
| MyHealthNB Access Format | 0.027 | 0.080 | 0.33 | .740 | [–0.13, 0.18] |
| **Combined Satisfaction Score** | **0.267** | 0.062 | **4.28** | **< .001** | **[0.14, 0.39]** |
| Home Page Use | 0.049 | 0.082 | 0.60 | .547 | [–0.11, 0.21] |
| MyHealthNB Use Frequency | 0.087 | 0.065 | 1.34 | .182 | [–0.04, 0.21] |
| *Satisfaction with Provider Encouragement* | *0.111* | 0.038 | *2.95* | *.003* | *[0.04, 0.18]* |

**Model for MyHealthNB’s Impact Visits to Providers**

**Model Summary**

R² = 0.117 Adjusted R² = 0.094

F (16, 602) = 4.99, p < .001

Standard Error = 0.88, n = 619

| **Predictor** | **B (Coeff.)** | **SE** | **t** | **p** | **95 % CI** |
| --- | --- | --- | --- | --- | --- |
| **Intercept** | **2.270** | 0.391 | **5.81** | **< .001** | **[1.50, 3.04]** |
| Age Range | –0.039 | 0.027 | –1.46 | .144 | [–0.09, 0.01] |
| Gender Identity | –0.166 | 0.085 | –1.95 | .051 | [–0.33, 0.00] |
| Racial Identity | –0.033 | 0.132 | –0.25 | .804 | [–0.29, 0.23] |
| Location Area | 0.167 | 0.104 | 1.61 | .109 | [–0.04, 0.37] |
| Residence Length | –0.017 | 0.046 | –0.37 | .709 | [–0.11, 0.07] |
| Language | –0.017 | 0.082 | –0.21 | .835 | [–0.18, 0.14] |
| Education Level | –0.031 | 0.088 | –0.35 | .730 | [–0.20, 0.14] |
| **Family Doctor** | **0.245** | 0.090 | **2.71** | **.007** | **[0.07, 0.42]** |
| Chronic Health | –0.102 | 0.089 | –1.14 | .253 | [–0.28, 0.07] |
| Web Confidence | 0.064 | 0.074 | 0.87 | .384 | [–0.08, 0.21] |
| Navigation Ease | 0.012 | 0.029 | 0.40 | .686 | [–0.05, 0.07] |
| MyHealthNB Access Format | 0.051 | 0.075 | 0.67 | .500 | [–0.10, 0.20] |
| **Combined Satisfaction Score** | **0.216** | 0.059 | **3.69** | **< .001** | **[0.10, 0.33]** |
| Home Page Use | 0.046 | 0.077 | 0.60 | .550 | [–0.10, 0.20] |
| *MyHealthNB Use Frequency* | *0.156* | 0.061 | *2.57* | *.010* | *[0.04, 0.28]* |
| *Satisfaction with Provider Encouragement* | *0.086* | 0.035 | *2.45* | *.015* | *[0.02, 0.16]* |

**Model for MyHealthNB’s Impact Visits to ER**

**Model Summary**
R² = 0.160 Adjusted R² = 0.138
F(16, 599) = 7.15, *p* < .001
Standard Error = 0.70, *n* = 616

| **Predictor** | **B (Coeff.)** | **SE** | **t** | **p** | **95 % CI** |
| --- | --- | --- | --- | --- | --- |
| **Intercept** | **2.537** | 0.312 | **8.12** | **< .001** | **[1.92, 3.15]** |
| Age Range | –0.011 | 0.021 | –0.50 | .618 | [–0.05, 0.03] |
| Gender Identity | –0.005 | 0.068 | –0.07 | .944 | [–0.14, 0.13] |
| Racial Identity | 0.071 | 0.105 | 0.67 | .501 | [–0.14, 0.28] |
| *Location Area* | *0.187* | 0.083 | *2.24* | *.025* | *[0.02, 0.35]* |
| **Residence Length** | **–0.098** | 0.037 | **–2.69** | **.007** | **[–0.17, –0.03]** |
| Language | 0.031 | 0.066 | 0.47 | .639 | [–0.10, 0.16] |
| *Education Level* | *–0.145* | 0.071 | *–2.05* | *.040* | *[–0.28, –0.01]* |
| *Family Doctor* | *0.148* | 0.072 | *2.07* | *.039* | *[0.01, 0.29]* |
| Chronic Health | –0.021 | 0.071 | –0.29 | .769 | [–0.16, 0.12] |
| Web Confidence | 0.009 | 0.059 | 0.15 | .882 | [–0.11, 0.12] |
| Navigation Ease | 0.015 | 0.023 | 0.62 | .533 | [–0.03, 0.06] |
| MyHealthNB Access Format | –0.029 | 0.060 | –0.48 | .629 | [–0.15, 0.09] |
| **Combined Satisfaction Score** | **0.139** | 0.047 | **2.97** | **.003** | **[0.05, 0.23]** |
| *Home Page Use* | *0.122* | 0.061 | *1.99* | *.047* | *[0.00, 0.24]* |
| *MyHealthNB Use Frequency* | *0.121* | 0.049 | *2.45* | *.014* | *[0.02, 0.22]* |
| **Satisfaction with Provider Encouragement** | **0.108** | 0.028 | **3.84** | **< .001** | **[0.05, 0.16]** |
